# Associations between long-term blood pressure trajectory and all-cause and CVD mortality among old people in China

**DOI:** 10.3389/fcvm.2023.1157327

**Published:** 2023-08-17

**Authors:** Huimeng Liu, Yutong Wang, Binyan Zhang, Jingchun Liu, Yating Huo, Suixia Cao, Shaowei Wu, Yong Wan, Xinming Xie, Lingxia Zeng, Hong Yan, Shaonong Dang, Baibing Mi

**Affiliations:** ^1^ Department of Occupational and Environmental Health, School of Public Health, Xi’an Jiaotong University Health Science Center, Xi’an, China; ^2^ Key Laboratory for Disease Prevention and Control and Health Promotion of Shaanxi Province, Xi’an, China; ^3^ Global Health Institute, School of Public Health, Xi’an Jiaotong University Health Science Center, Xi’an, China; ^4^ Department of Epidemiology and Biostatistics, School of Public Health, Xi’an Jiaotong University Health Science Center, Xi’an, China; ^5^ Department of Geriatric Surgery, First Affiliated Hospital, Xi’an Jiaotong University, Xi’an, China; ^6^ Department of Respiratory and Critical Care Medicine, First Affiliated Hospital of Xi’an Jiaotong University, Xi'an, China

**Keywords:** blood pressure trajectory, the elderly, all-cause mortality, CVD mortality, blood pressure trajectory before death

## Abstract

**Background:**

Optimal blood pressure (BP) management strategy among the elderly remains controversial, with insufficient consideration of long-term BP trajectory. This study aimed to identify BP trajectory patterns as well as terminal BP trajectory among the Chinese elderly and to explore the relationships between BP trajectories and all-cause mortality and cardiovascular disease (CVD) mortality.

**Methods:**

We included 11,181 participants older than 60 at baseline (mean age, 80.98 ± 10.71) with 42,871 routine BP measurements from the Chinese Longitudinal Healthy Longevity Survey. Latent class trajectory analysis and Cox proportional hazard model were conducted to identify trajectory patterns and their associations with mortality. Furthermore, we also applied mixed-effects model to identify terminal BP trajectories among the elderly.

**Results:**

Compared with stable at normal high level trajectory, excess systolic BP (SBP) trajectory with decreasing trend was associated with a 34% (HR = 1.34, 95% CI: 1.23–1.45) higher risk of all-cause mortality. Considering the competing risk of non-CVD death, excess BP trajectory with decreasing trend had a more pronounced effect on CVD mortality, in which HR (95% CI) was 1.67 (1.17, 2.37). Similar results were also found in diastolic BP (DBP), pulse pressure (PP), and mean arterial pressure (MAP) trajectories. We further conducted a mixed-effects model and observed that SBP and PP trajectories first increased and began to decline slightly six years before death. In contrast, DBP and MAP showed continuous decline 15 years before death.

**Conclusion:**

Long-term BP trajectory was associated with all-cause mortality, especially CVD mortality. Keeping a stable BP over time may be an important way for CVD prevention among the elderly.

## 1. Introduction

Population aging has been recognized as one of the major challenges globally, including in China (1, 2). During the aging period, blood pressure (BP) dynamically changes during the aging process because of arterial stiffness and other age-related variation of blood vessels (3). It has been testified that these age-related BP changes were potent determinants of cardiovascular disease (CVD) events and all-cause mortality (3). Dynamic BP management among the elderly, especially very old people, may be an effective strategy for reducing the risks of adverse health outcomes.

Long-term repeated BP measurements can better reveal the potential causation of cardiovascular disease's pathophysiological progression, but to date, most research has focused only on a single measurement. BP trajectory, usually identified by the latent class model, may provide additional value to CVD risk prediction and new sights in assessing life span BP variation because it can reflect long-term changes of individual BP over time and consider lifetime patterns such as starting levels, slope, and cumulative exposure (4, 5). Nevertheless, a majority of research considering BP trajectories was restricted to limited inclusion of the oldest-old participants or centenarians; therefore, most of those BP trajectories were at a high level or showed increasing patterns (6–11). For the very old elderly (usually older than 80 years old), terminal decline of BP is typical and has implications for risk assessment, BP monitoring, and study design (9). However, long-term BP trajectories and their patterns during the terminal period are little known among the Chinese elderly.

Regarding the relationship between BP and all-cause mortality among the elderly, recent studies left us with more confusion. Strategy of Blood Pressure Intervention in the Elderly Hypertensive Patients (STEP) found that intensive BP treatment, which controlled SBP at the range of 110–130 mmHg had no effect on the risk of all-cause mortality (hazard ratio = 1.11, 95% CI: 0.78–1.56) (12), while in Systolic Blood Pressure Intervention Trial (SPRINT), impressive benefits on death from any cause was observed (hazard ratio = 0.75, 95% CI: 0.61–0.92) in intensive treatment groups, in which target SBP was no more than 120 mmHg (13). It is worth mentioning that we should be more cautious when generalizing the results from SPRINT. For the elderly whose life expectancy is less than one year, with low Framingham risk score and high baseline SBP (at least 160 mmHg), intensive SBP treatment may not be a good choice (14, 15). Some observational studies suggested the cautious application of SBP less than 130 mmHg and observed a U or J shape relationship between BP and all-cause mortality (16, 17). These findings contradicted the hypothesis of “low the best” among the elderly and may somewhat explain the inconsistency between clinical trials and observational studies. Thus, there would seem to be a definite need for seeking further evidence on more consideration of the elderly health status and long-term trajectories of BP when making optimal management strategies.

Our study, based on the Chinese Longitudinal Healthy Longevity Survey (CLHLS), which could highly represent the oldest people in China (2), aimed to identify long-term BP trajectory as well as terminal BP trajectory. And further explore the associations of BP trajectories with all-cause mortality and CVD mortality among the Chines elderly who died after 60 years old.

## 2. Methods

### 2.1. Study sample

CLHLS is a nationwide, longitudinal cohort study covering half of the counties and cities randomly selected from 22 provinces in China. So far, it has conducted eight survey waves in 1998, 2000, 2002, 2005, 2008, 2011, 2014, and 2018 with high quality and representativeness (2). More details about the study design can be found elsewhere (18, 19). After death or loss to follow-up, new participants would be enrolled and matched with the same gender and age nearby (20). In the current study, 56,949 subjects from 8 waves of the CLHLS from 1999 to 2018 were initially included. We excluded participants with the following conditions: younger than 60 years old at baseline (*N* = 168), had missing data on the investigation date (*N* = 390), had outliers of BP measurements (systolic BP (SBP) < 40 mmHg or SBP > 250 mmHg; diastolic BP (DBP) < 40 mmHg or DBP >140, *N* = 2,115), had BP measurements less than three times during the follow-up period (*N* = 42,925) or had missing data on accurate death date (*N* = 170). Finally, 11,181 participants older than 60 years at baseline were involved in the subsequent analysis (Supplementary Figure S1).

The CLHLS study was approved by the Biomedical Ethics Committee of Peking University, Beijing, China (IRB00001052-13074), and all participants or their proxy respondents have informed consent.

### 2.2. BP measurement and trajectories

BP measurements were conducted according to standard protocols by well-trained interviewers. Participants were measured for BP twice on the right arm by mercury sphygmomanometer (upper arm type; Yuyue, Jiangsu, China) after at least five minutes of rest. Bedbound participants were measured in a recumbent position. The mean value of the two measurements was used in further analysis. Pulse pressure (PP) was calculated as SBP-DBP, and mean arterial pressure (MAP) was calculated as (SBP + 2*DBP)/3 (16).

**Figure 1:**
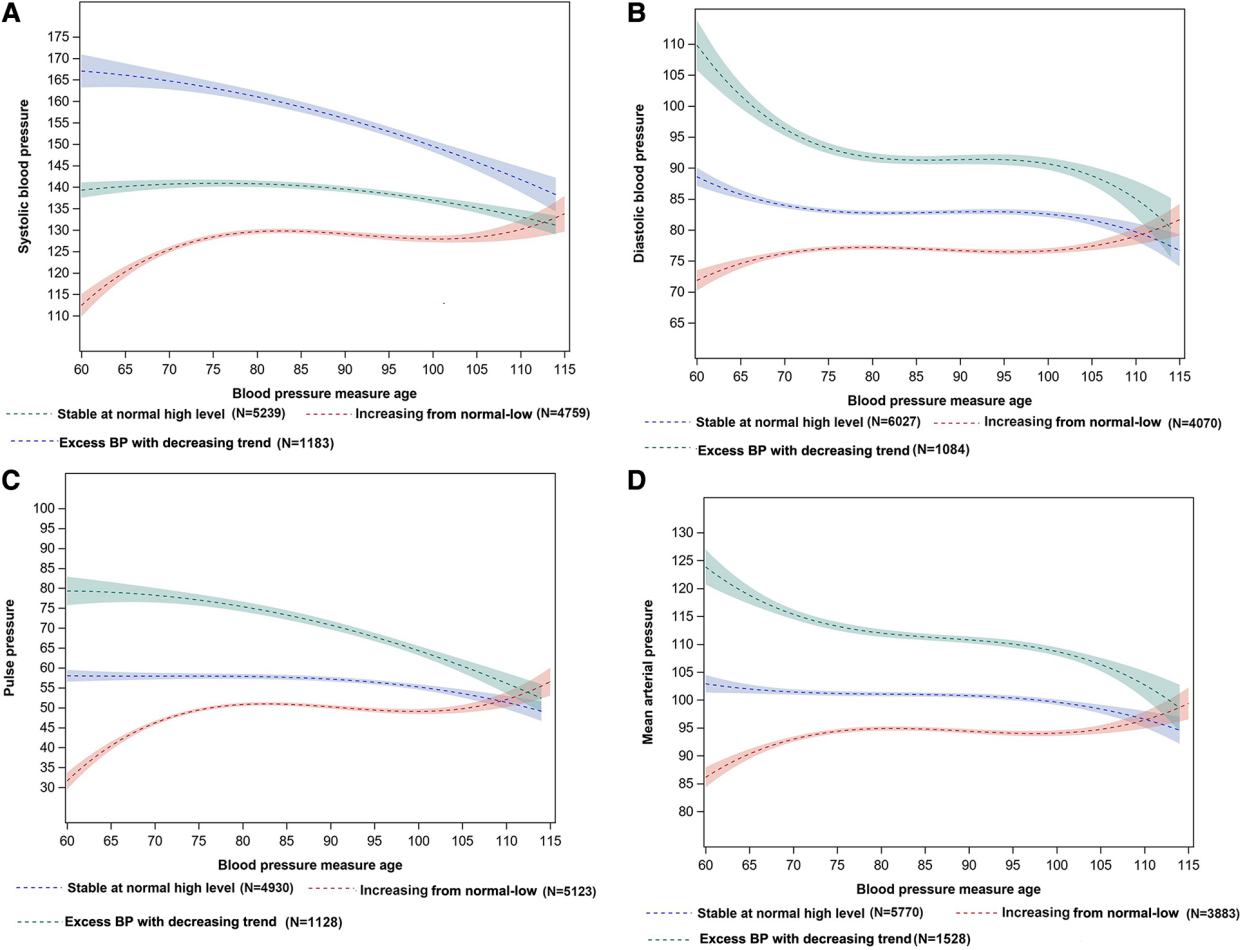
BP trajectories of SBP (**A**), DBP (**B**), PP (**C**), and MAP (**D**) with measure age among the Chinese elderly. (**A**) Systolic blood pressure (SBP), (**B**) Diastolic blood pressure (DBP), (**C**) Pulse pressure (PP), (**D**) Mean arterial pressure (MAP). The shaded area represents 95%CI of the BP trajectories.

Given the dynamic BP changes among the elderly (9), there was a conceptual rationale to examine BP trajectories with age. We assumed that long-term BP change had several different and unobserved trajectories in the current study. Then, we used latent class trajectory analysis (LCTA) to identify long-term BP in a censored normal model with baseline BP as the risk factor (21). The procedure of trajectory identification was described in Supplementary Method S1. We tested the number of trajectory groups from 2–5, and used the following criteria to determine the optimal number of groups, and polynomial type (intercept, linear, or quadratic) of each trajectory: (1) satisfies Bayesian Information Criterion (BIC), (2) each trajectory contains at least 5% predicted sample size, (3) average posterior probability greater than 70%, and (4) have the clinical meaning of each trajectory (21, 22). Finally, trajectory membership was recognized as an independent variable in the subsequent analyses. The fit indices and parameter estimation of 2–5 groups for BP trajectories were presented in Supplementary Tables S1, S2. Finally, three groups' distinct trajectories were identified in SBP, DBP, PP, and MAP (Figure 1).

### 2.3. Confounders assessment

Potential confounders were identified based on existing literature rather than deferring to statistical criteria (23, 24). We assessed demographic characteristics, socio-economic status, lifestyle behavior change, and health status (25, 26), which were reported from face-to-face interviews during each follow-up survey. Demographic characteristics and socio-economic variables included sex (male/female), ethnicity (Han nationality/others), average household income (<5,000/5,000–19,999/>20,000 yuan), place of residence (city/town/rural), main occupation before retired (professional and technical personnel governmental/institutional or managerial personnel/agriculture, forest, animal husbandry/fishery worker/industrial worker/ commercial or service worker/military personnel/housework/others), marriage status (currently married and living with spouse/separated/divorced/widowed/never married). Lifestyle behavior changes included regular exercise (yes/no), drinking, and smoking status, which were categorized according to the transition between the first visit and the last visit (non-smoker or drinker/ex-smoker or drinker/always smoking or drinking/current smoker or drinker). Health status included comorbidity and frail status. The current study defined comorbidity as the number of having the following chronic diseases during the follow-up period: hypertension, diabetes, coronary heart disease, cardiovascular disease, pulmonary diseases, tuberculosis, cancer, tumor, and Parkinson's disease. Frailty Index (FI), including 35 health deficits, was assessed at the first visit and calculated using the cumulative deficits-based standard method (27). More details can be found in Supplementary Method S2. Furthermore, the frail status was categorized into non-frail for those with FI ≤ 0.1, pre-frail for 0.1 < FI ≤ 0.21, and frail for FI > 0.21 (28). Because BP trajectories were identified over measurement age, we did not adjust for age further in the model. All confounders, except for behavior change, were assessed from the first interview.

### 2.4. Ascertainment of death

Participants' survival status was collected at each follow-up survey. Before the 2018 wave, for those who were interviewed in the previous wave, but died before the next survey, the death date and death cause were ascertained by the decedents' next-of-kin or village doctors (10). In the 2018 wave, the cause of death was ascertained during the follow-up survey in 2017–2018, and cardiovascular mortality was ascertained by ICD-10 (international classification of diseases, 10th revision) codes of I00-I99 (29, 30). The primary outcome was total mortality, and the secondary outcomes were death from CVD or non-CVD.

### 2.5. Statistical analysis

Demographic and health-related characteristics of the study participants were described across BP trajectories and presented as mean (standard derivation) for continuous variables and numbers (percentages) for categorical variables. ANOVA tested variance across BP trajectories for continuous variables and $\chi^{2}$ test for categorical variables, respectively.

We used the Cox proportional hazard regression model to explore the associations between BP trajectories and the risk of all-cause mortality. For decedents, survival time was the study enrollment period to the date of death. Survival time was calculated from the enrollment date to the last visit date for survivors or censored people. We established four models to quantitatively assess the effect of BP trajectories on all-cause mortality. Model 1 was the crude model without confounder adjustment. Model 2 adjusted for demographic and socio-economic characteristics of sex, ethnicity, baseline marriage status, baseline place of residence, main occupation before retirement, and average family income. In model 3, we additionally adjusted for lifestyle behaviors and health status including exercise at baseline, smoking status, drinking status, comorbidity status, and baseline frail status. Model 4 was additionally adjusted for baseline BP to identify the independent association between BP trajectories and mortality. Competing risk analysis with the cause-specific hazard model was used to compare the association with BP trajectories among CVD mortality and non-CVD mortality (31). To further explore the BP trajectory before death, we conducted a mixed-effects model with random intercept among decedents after adjusting for sex, BP measure age, and calendar year of the survey wave. The mixed-effects model accommodates the complexities of typical longitudinal data sets and is a valuable tool for explicating variation between and within subjects (32). Under BIC, we selected the best-fitted model from three mixed-effects models with linear, quadratic, and cubic items to identify the BP trajectories before death. We only included those who died within 15 years after enrollment in the study because of data availability. Detailed procedure and model selection were described in Supplementary Method S3.

Furthermore, we did analyses across sex, baseline place of residence, baseline age, smoking status, drinking status, frail status, and comorbidity subgroups. Sensitivity analyses included: (1) analyses on excluding bedridden participants, (2) multivariate imputation by chained equations (MICE) to reduce selection bias due to missing data on confounders (33) (more detailed information on multiple imputations can be found in Supplementary Method S4).

All statistical tests were two-sided, and statistical significance was defined as *P *< 0.05. The analyses were conducted using SAS 9.4 (SAS Institute, Cary, NC, USA).

## 3. Results

### 3.1. The patterns of BP trajectories and baseline characteristics

A total of 42,871 BP measurements were used to identify BP trajectories. Three SBP trajectories were identified (Figure 1A) based on BP levels and long-term dynamic changing trends. The first trajectory, characterized by stability at the normal high level of about 140 mmHg, was named the “stable at normal high level” trajectory (*n* = 5,239, 46.86%). The second trajectory, characterized by an increasing trend from 110 to 135 mmHg, was named the “increasing from normal-low” trajectory (*n* = 4,759, 42.56%). The third trajectory, characterized by constantly exceeding the normal BP range with decreasing trend from 170 to 140 mmHg was named the “excess BP trajectory with decreasing trend” (*n* = 1,183, 10.58%). The same trajectory pattern was also observed in DBP, PP, and MAP (Figure 1).

Compared with participants with a stable SBP trajectory at a normal high level, those who had an excess BP trajectory with decreasing trend were older, contained more females, had lower household income, included more non-smokers, and more non-drinkers, more frail people, and had more comorbidities (Table 1). Characteristics across DBP, PP, and MAP trajectories were similar to the SBP trajectories Supplementary Tables S7–S9).

**Table 1:** Demographic characteristics of the study participants by SBP trajectories.

Characteristics	Stable at normal high level	Increasing from normal-low	Excess BP trajectory with decreasing trend	*P-*value^a^
Number of participants (%)	5,239 (46.86)	4,759 (42.56)	1,183 (10.58)	* *
BP measurements *n* (%)	19,926 (46.48)	18,749 (43.73)	4,196 (9.79)	* *
Mean baseline age (SD)	81.06 (10.50)	79.76 (10.89)	84.68 (9.96)	**<0.001**
Sex *n* (%)				
Male	2,428 (46.34)	2,236 (46.98)	456 (38.55)	**<0.001**
Female	2,811 (53.66)	2,523 (53.02)	727 (61.45)	
Ethnicity *n* (%)				
Han nationality	2,201 (94.30)	7,118 (92.63)	984 (94.80)	**<0.001**
Others	275 (5.98)	335 (8.05)	54 (5.20)	
Average household income (yuan) *n* (%)				
Less than 5,000	1,650 (31.50)	1,263 (26.54)	464 (39.22)	**<0.001**
5,000–19,999	1,598 (30.51)	1,547 (32.51)	356 (30.09)	
More than 20,000	1,990 (37.99)	1,948 (40.94)	363 (30.68)	
Baseline place of residence *n* (%)				
City	1,223 (23.34)	1,005 (21.12)	327 (27.64)	**<0.001**
Town	1,673 (31.93)	1,167 (24.52)	471 (39.81)	
Rural	2,343 (44.72)	2,587 (54.36)	385 (32.54)	
Main occupation before retirement *n* (%)				
Professional and technical personnel	222 (4.25)	247 (5.20)	70 (5.93)	**<0.001**
Governmental, institutional, or managerial personnel	195 (3.73)	170 (3.58)	37 (3.14)	
Agriculture, forest, animal husbandry	1,487 (28.48)	1,135 (23.91)	382 (32.37)	
Fishery worker	72 (1.38)	75 (1.58)	13 (1.10)	
Industrial worker	2,376 (45.51)	2,410 (50.77)	444 (37.63)	
Commercial or service worker	421 (8.06)	326 (6.87)	91 (7.71)	
Military personnel	31 (0.59)	32 (0.67)	8 (0.68)	
Housework	339 (6.49)	243 (5.12)	110 (9.32)	
Others	74 (1.42)	96 (2.02)	22 (1.86)	
Marriage at baseline *n* (%)				
Currently married and living with spouse	2,088 (39.88)	1,953 (41.05)	384 (32.49)	**<0.001**
Separated	100 (1.91)	122 (2.56)	17 (1.44)	
Divorced	21 (0.40)	30 (0.63)	7 (0.59)	
Widowed	2,960 (56.53)	2,599 (54.62)	762 (64.47)	
Never married	67 (1.28)	54 (1.13)	12 (1.02)	
Smoking status *n* (%)				
Non-smokers	3,874 (74.04)	3,437 (72.34)	936 (79.39)	**<0.001**
Ex-smokers	543 (10.38)	496 (10.44)	108 (9.16)	
Always smoking	643 (12.29)	639 (13.45)	113 (9.58)	
Current smoker	172 (3.29)	179 (3.77)	22 (1.87)	
Drinking status *n* (%)				
Non-drinkers	3,707 (70.88)	3,311 (69.76)	888 (75.32)	**0.026**
Ex-drinkers	701 (13.40)	659 (13.89)	133 (11.28)	
Always drinking	550 (10.52)	511 (10.77)	105 (8.91)	
Current drinker	272 (5.20)	265 (5.58)	53 (4.50)	
Exercise at baseline *n* (%)				
Yes	1,799 (34.35)	1,670 (35.10)	395 (33.39)	0.499
No	3,438 (65.65)	3,088 (64.90)	788 (66.61)	
Frailty status *n* (%)				
No frail	2,889 (55.14)	3,069 (64.49)	465 (39.31)	**<0.001**
Pre frail	1,979 (37.77)	1,445 (30.36)	572 (48.35)	
Frail	371 (7.08)	245 (5.15)	146 (12.34)	
Comorbidity^b^ *n* (%)				
No comorbidity	1,314 (25.08)	1,619 (34.02)	156 (13.19)	**<0.001**
1	1,694 (32.33)	1,581 (33.22)	428 (36.18)	
2	1,208 (23.06)	894 (18.79)	344 (29.08)	
More than and equal to 3	1,023 (19.53)	665 (13.97)	255 (21.56)	
CVD death *n* (%)	152 (2.90)	114 (2.40)	55 (4.65)	**<0.001**

CVD, cardiovascular disease; SBP, systolic blood pressure; SD, standard deviation.

*P*-value <0.05 was highlighted in bold.

^a^ ANOVA was used to test variance across BP trajectories for continuous variables and *χ*^2^ test for categorical variables.

^b^ Number of following chronic diseases during the follow-up period: hypertension, diabetes, coronary heart disease, cardiovascular disease, pulmonary diseases, tuberculosis, cancer, tumor, Parkinson.

### 3.2. Associations between BP trajectories and all-cause mortality

During the median follow-up of 8.4 (interquartile range, 6.1–11.0) years, 6,010 death occurred (mean death age 85.26 ± 9.66), and the overall death rate was 6.01 per 100 person-years. Supplementary Figure S2 demonstrated the cumulative survival rates of SBP, DBP, PP, and MAP trajectories. Cumulative survival probability was the lowest for those who had excess BP trajectories with decreasing trends. The results were similar for DBP, PP, and MAP trajectories. Table 2 showed the multi-variable adjusted association between BP trajectories and all-cause mortality. Compared with the stable at normal high level trajectory, excess SBP trajectory with decreasing trend was associated with a 63% higher risk of all-cause mortality (HR = 1.63, 95% CI: 1.50–1.77) in the crude model, and this ratio was 34% [adjusted HR (aHR) = 1.34, 95% CI: 1.23–1.45] in model 3. The same results were also observed in the excess DBP, PP, and MAP trajectories with decreasing trend, which was associated with a 15% (aHR = 1.15, 95% CI: 1.06–1.25), 30% (aHR = 1.30, 95% CI: 1.19–1.42), and 27% (aHR = 1.27, 95% CI: 1.17–1.37) higher risk of all-cause mortality, respectively. Meanwhile, we observed a protective effect of increasing from normal-low trajectories (aHR = 0.87, 95% CI: 0.82–0.92 for SBP trajectory, aHR = 0.93, 95% CI: 0.88–0.98 for DBP trajectory, aHR = 0.89, 95% CI: 0.84–0.94 for PP trajectory, and aHR = 0.91, 95% CI: 0.86–0.96 for MAP trajectory) on all-cause mortality when compared with the stable at normal high-level trajectory. After additionally adjusting for baseline BP, the result remained unchangeable.

**Table 2:** Association of blood pressure trajectory and all-cause mortality.

Blood pressure trajectories	Model 1^a^ HR (95%CI)	*P*-value	Model 2^b^ HR (95%CI)	*P*-value	Model 3^c^ HR (95%CI)	*P*-value	Model 4^d^ HR (95%CI)	*P*-value
SBP trajectory								
Stable at normal high level	Reference	Reference	Reference	Reference	Reference	Reference	Reference	Reference
Increasing from normal-low	**0.81** **(0.77,0.86)**	**<0** **.** **001**	**0.90** **(0.86,0.96)**	**<0** **.** **001**	**0.87** **(0.82,0.92)**	**<0** **.** **001**	**0.85** **(0.78,0.92)**	**<0** **.** **001**
Excess SBP trajectory with decreasing trend	**1.63** **(1.50,1.77)**	**<0** **.** **001**	**1.32** **(1.22,1.44)**	**<0.001**	**1.34** **(1.23,1.45)**	**<0** **.** **001**	**1.39** **(1.23,1.55)**	**<0** **.** **001**
DBP trajectory								
Stable at normal high level	Reference	Reference	Reference	Reference	Reference	Reference	Reference	Reference
Increasing from normal-low	**0.89** **(0.84,0.94)**	**<0** **.** **001**	0.96 (0.90,1.01)	0.116	**0.93** **(0.88,0.98)**	**0** **.** **008**	**0.78** **(0.72,0.85)**	**<0** **.** **001**
Excess SBP trajectory with decreasing trend	**1.29** **(1.19,1.41)**	**<0** **.** **001**	**1.15** **(1.06,1.26)**	**0** **.** **001**	**1.15** **(1.06,1.25)**	**0** **.** **002**	**1.46** **(1.30,1.63)**	**<0** **.** **001**
PP trajectory								
Stable at normal high level	Reference	Reference	Reference	Reference	Reference	Reference	Reference	Reference
Increasing from normal-low	**0.78** **(0.74,0.82)**	**<0** **.** **001**	**0.89** **(0.85,0.94)**	**<0** **.** **001**	**0.89** **(0.84,0.94)**	**<0** **.** **001**	0.98 (0.90,1.06)	0.600
Excess SBP trajectory with decreasing trend	**1.48** **(1.36,1.62)**	**<0** **.** **001**	**1.28** **(1.17,1.40)**	**<0** **.** **001**	**1.30** **(1.19,1.42)**	**<0** **.** **001**	**1.15 (1.02,1.30)**	**0** **.** **024**
MAP trajectory								
Stable at normal high level	Reference	Reference	Reference	Reference	Reference	Reference	Reference	Reference
Increasing from normal-low	**0.88** **(0.84,0.94)**	**<0** **.** **001**	**0.94** **(0.89,0.99)**	**0** **.** **027**	**0.91** **(0.86,0.96)**	**0** **.** **001**	**0.81** **(0.75,0.88)**	**<0** **.** **001**
Excess SBP trajectory with decreasing trend	**1.49** **(1.39,1.61)**	**<0** **.** **001**	**1.24** **(1.15,1.34)**	**<0** **.** **001**	**1.27** **(1.17,1.37)**	**<0** **.** **001**	**1.48** **(1.33,1.65)**	**<0** **.** **001**

SBP, systolic blood pressure; DBP, diastolic blood pressure; PP, pulse pressure; MAP, mean arterial pressure.

^a^ Model 1 was the crude model.

^b^ Model 2 was adjusted for sex, ethnicity, baseline marriage status, baseline place of residence, main occupation before retirement, and average family income.

^c^ Model 3 was additionally adjusted for exercise at baseline, smoking status, drinking status, comorbidity status, and frail status based on Model 2.

^d^ Model 4 was additionally adjusted for baseline BP based on Model 3.

*P*-value <0.05 was highlighted in bold.

**Figure 2:**
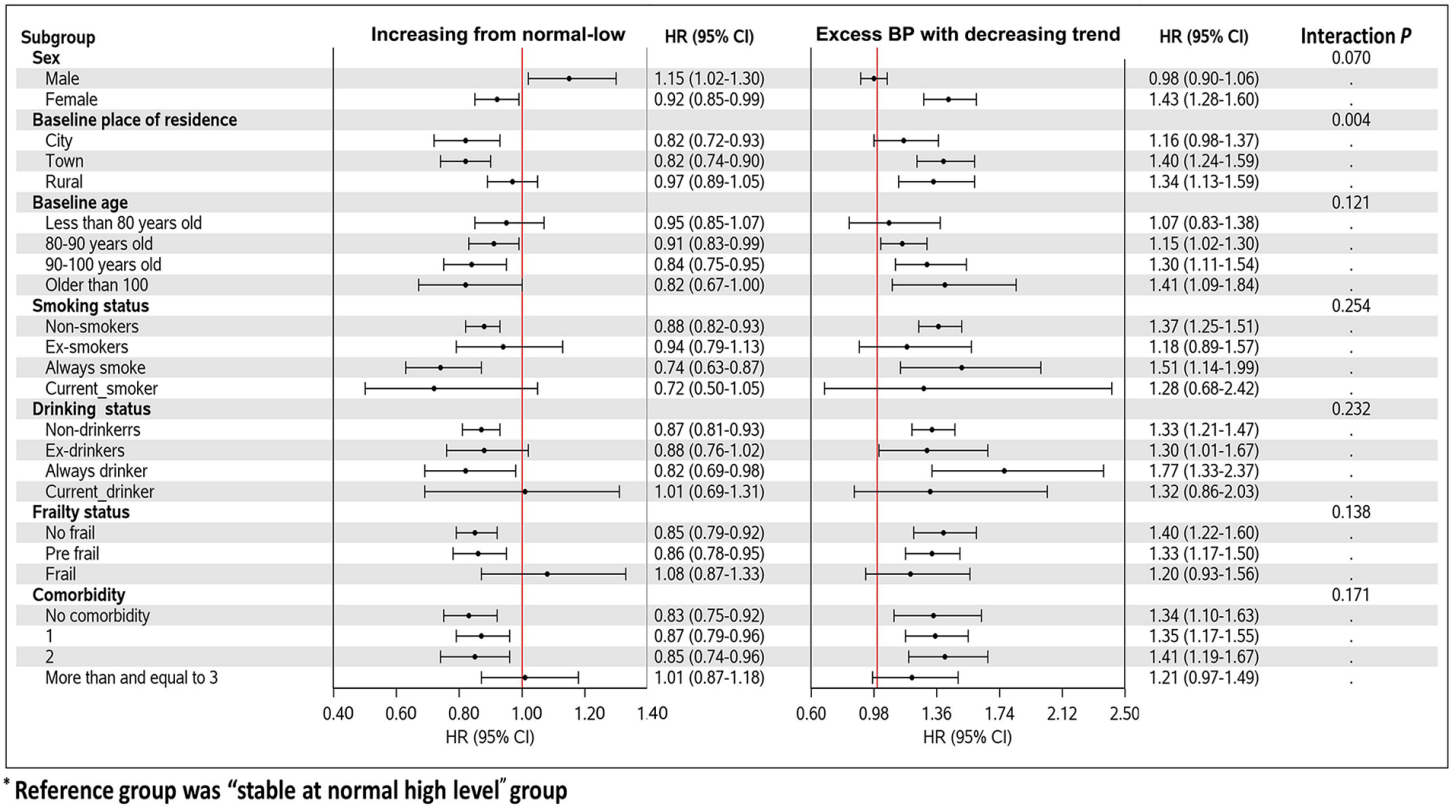
Forest plot of the associations with SBP trajectories and mortality in different subgroups.

In subgroup analyses for SBP trajectory, a significant interaction with SBP trajectory was only found in the subgroup of living areas (*P* for interaction = 0.004, Figure 2), which suggested that the associations between SBP trajectories and the risk of all-cause mortality were consistent in other subgroups. The associations of DBP, PP, and MAP trajectories and all-cause mortality by subgroups were shown in Supplementary Figure S3.

### 3.3. Associations between BP trajectories and CVD mortality

In this study, we observed 321 (2.5%) deaths attributed to CVD. Survival status by sex, age, and ethnic groups was presented in Supplementary Table S10. It showed that the survival status had no variance among sex, and the prevalence of CVD mortality was higher among the older participants and Han participants. We further considered the competing risk of non-CVD death when investigating the associations between BP trajectories and CVD mortality (Table 3). Compared with the stable trajectory, excess BP trajectory with decreasing trend increased the cause-specific hazard of CVD death by 67%, and non-CVD death by 32%, which showed a more pronounced effect on the cause-specific hazard of CVD death than non-CVD death. The same results were found in PP, and MAP trajectories. However, after adjusting for baseline BP, the associations between BP trajectories and CVD mortality became insignificant, except for PP trajectories.

**Table 3:** Hazard ratios (and 95% confidence intervals) from cause-specific hazard models for CVD and non-CVD mortality.

Blood pressure trajectories	Model 1^a^ HR (95%CI)		Model 2^b^ HR (95%CI)		Model 3^c^ HR (95%CI)		Model 4^d^ HR (95%CI)	
	CVD mortality	Non-CVD mortality	CVD mortality	Non-CVD mortality	CVD mortality	Non-CVD mortality	CVD mortality	Non-CVD mortality
SBP trajectory								
Stable at normal high level	Reference	Reference	Reference	Reference	Reference	Reference	Reference	Reference
Increasing from normal-low	**0.68** **(0.53,0.87)**	**0.82** **(0.78,0.87)**	**0.76** **(0.59,0.98)**	**0.91** **(0.86,0.97)**	0.81 (0.63,1.05)	**0.87** **(0.82,0.92)**	0.88 (0.61,1.25)	**0.85** **(0.78,0.92)**
Excess SBP trajectory with decreasing trend	**2.35** **(1.72,3.20)**	**1.59** **(1.46,1.73)**	**1.79** **(1.26,2.53)**	**1.30** **(1.19,1.42)**	**1.67** **(1.17,2.37)**	**1.32** **(1.21,1.44)**	1.49 (0.91,2.44)	**1.38** **(1.22,1.55)**
DBP trajectory								
Stable at normal high level	Reference	Reference	Reference	Reference	Reference	Reference	Reference	Reference
Increasing from normal-low	0.86 (0.67,1.09)	**0.89** **(0.84,0.94)**	0.95 (0.73,1.22)	0.96 (0.90,1.01)	0.95 (0.74,1.23)	**0.93** **(0.87,0.98)**	0.86 (0.61,1.22)	**0.78** **(0.72,0.84)**
Excess SBP trajectory with decreasing trend	**1.54** **(1.09,2.16)**	**1.28** **(1.17,1.39)**	**1.53** **(1.07,2.18)**	**1.13** **(1.04,1.24)**	1.32 (0.92,1.90)	**1.14** **(1.04,1.25)**	1.53 (0.93,2.50)	**1.45** **(1.29,1.63)**
PP trajectory								
Stable at normal high level	Reference	Reference	Reference	Reference	Reference	Reference	Reference	Reference
Increasing from normal-low	**0.59** **(0.46,0.75)**	**0.79** **(0.75,0.83)**	**0.66** **(0.52,0.85)**	**0.91** **(0.86,0.96)**	**0.70** **(0.54,0.90)**	**0.90** **(0.85,0.96)**	**0.62** **(0.43,0.89)**	1.00 (0.92,1.09)
Excess SBP trajectory with decreasing trend	**2.30** **(1.68,3.16)**	**1.44** **(1.32,1.57)**	**1.73** **(1.20,2.48)**	**1.26** **(1.15,1.38)**	**1.70** **(1.18,2.45)**	**1.28** **(1.16,1.40)**	**2.00** **(1.21,3.31)**	1.11 (0.98,1.26)
MAP trajectory								
Stable at normal high level	Reference	Reference	Reference	Reference	Reference	Reference	Reference	Reference
Increasing under control	**0.76** **(0.59,0.98)**	**0.89** **(0.84,0.94)**	0.85 (0.65,1.11)	**0.94** **(0.89,1.00)**	0.87 (0.67,1.14)	**0.91** **(0.86,0.97)**	0.85 (0.59,1.21)	**0.81** **(0.74,0.88)**
Uncontrolled excess MAP trajectory with decreasing trend	**1.98** **(1.48,2.64)**	**1.47** **(1.36,1.58)**	**1.67** **(1.22,2.30)**	**1.22** **(1.13,1.32)**	**1.49** **(1.07,2.06)**	**1.25** **(1.16,1.36)**	1.54 (0.97,2.45)	**1.47** **(1.32,1.65)**

SBP, systolic blood pressure; DBP, diastolic blood pressure; PP, pulse pressure; MAP, mean arterial pressure.

*P*-value <0.05 was highlighted in bold.

^a^ Model 1 was the crude model.

^b^ Model 2 was adjusted for sex, ethnicity, baseline marriage status, baseline place of residence, main occupation before retirement, and average family income.

^c^ Model 3 was additionally adjusted for exercise at baseline, smoking status, drinking status, comorbidity status, and frail status based on Model 2.

^d^ Model 4 was additionally adjusted for baseline BP based on Model 3.

### 3.4. BP trajectories 15 years before death

**Figure 3:**
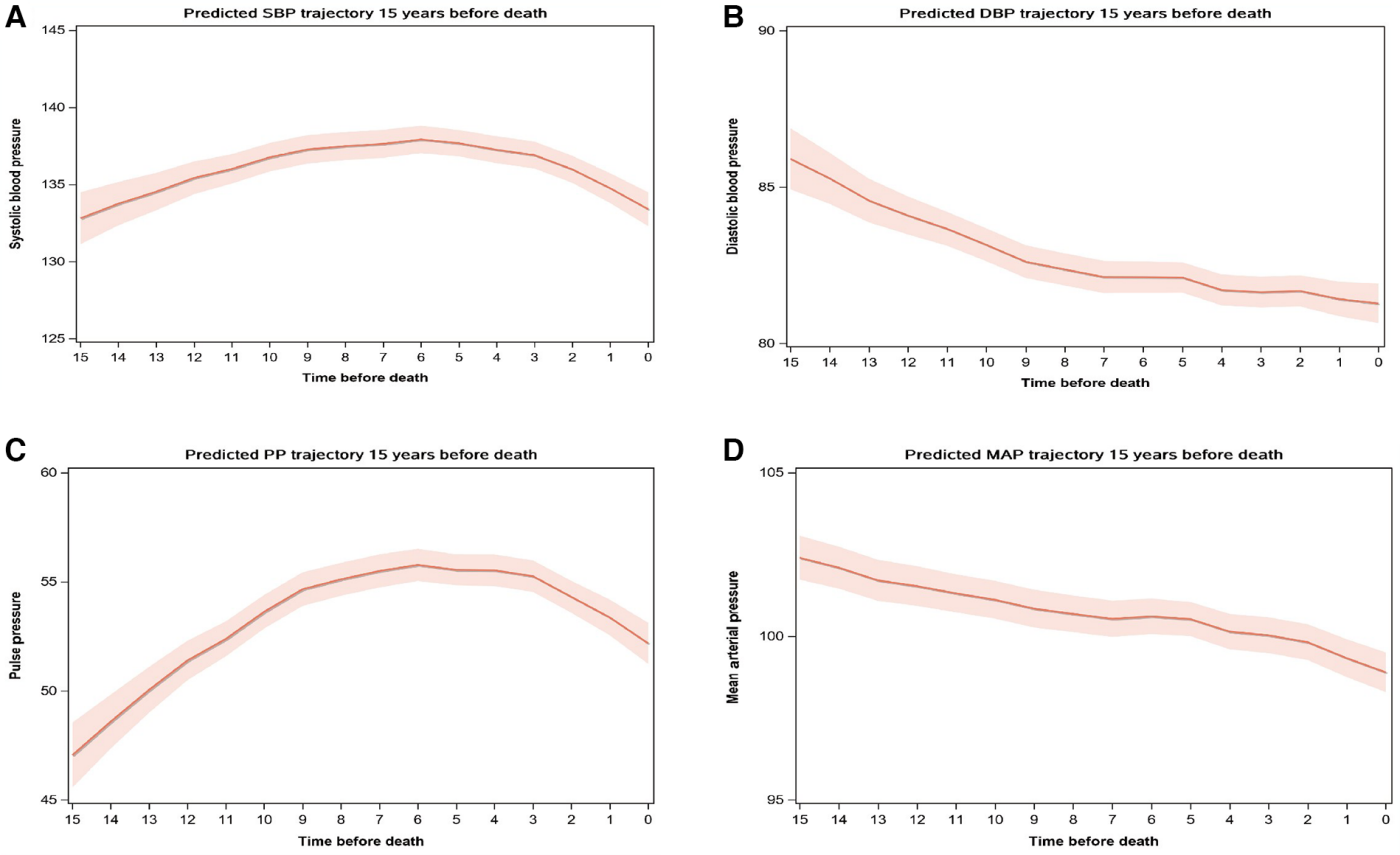
BP trajectories 15 years before death. *(**A**) SBP trajectory, (**B**) DBP trajectory, (**C**) PP trajectory, (**D**) MAP trajectory. ^†^Mixed-effects model with random intercept was used to estimate BP trajectory 15 years before death among the decedents after adjusting for sex, BP measure age, and calendar year of the survey wave. SBP, Systolic blood pressure; DBP, diastolic blood pressure; PP, pulse pressure; MAP, mean arterial pressure.

Among 6,010 decedents, we further explored the 15-year BP trajectories prior to death by a mixed-effects model (Figure 3). SBP and PP represented similar trajectories with a slight increase and then began to decline six years before death. SBP trajectories presented the same trend in different sex, frail status, and death age groups (Supplementary Figures S4–S6). We also observed that, for those who died older than 100 years old, the estimated 15-year SBP trajectories before death were less fluctuating, consistent with our results showing a beneficial effect of stable BP for all-cause mortality. In addition, DBP and MAP showed approximately linear decline 15 years before death.

### 3.5. Sensitivity analyses

Sensitivity analyses tested the robustness of the associations between uncontrolled BP trajectories and all-cause mortality and CVD mortality. After excluding bedridden participants and conducting multiple imputations, we observed similar results, which showed the robustness of our study (Supplementary Tables S11, S12).

## 4. Discussion

In this nationwide prospective cohort study among the elderly in China, we found that long-term excess BP trajectory with decreasing trend was associated with a higher risk of all-cause mortality and CVD mortality compared with stable at normal high level trajectory. Similar results were also found in DBP, PP, and MAP trajectories. Furthermore, for the decedents, we observed that SBP and PP trajectories increased and then declined six years before death slightly. While DBP and MAP trajectories showed continuous decline 15 years prior to death. These results indicated that long-term excess BP trajectory with decreasing trend might be a risk factor for death from any cause, especially death caused by CVD. Meanwhile, independent associations were observed among excess BP trajectory with decreasing trend and all-cause mortality regardless of baseline BP levels. The attention to long-term BP trajectories should be raised among the elderly.

### 4.1. Associations between BP trajectories and risk of mortality

Till now, randomized clinical trials (RCTs) and observational studies have given different answers to BP control strategy among the oldest people and have not reached an agreement yet (17, 34). Unlike current studies, our results focused on the long-term BP trajectories and provided evidence of a long-term dynamic BP monitoring strategy. The associations between excess BP with decreasing trend and mortality might be attributed to lasting excess BP exposure, which has been well explored in previous studies. Analysis based on two extensive cohort studies (Health and Retirement Study, median age: 65 years old, and English Longitudinal Study of Ageing, median age: 62 years old) reported that larger cumulative SBP and PP were independently associated with faster cognitive decline and higher all-cause mortality (35). As for single measurement, a nationwide population-based cohort study included 374,250 participants older than 75 years old in Korea found that SBP higher than 170 mmHg had a 17% (HR:1.17, 95% CI: 1.13–1.21) higher risk of mortality compared with SBP at 120–129 mmHg (17). A community-dwelling octogenarians' study in Shanghai also found that compared with SBP at 117–145 mmHg, higher SBP was associated with a 34% higher risk of all-cause mortality (36).

As for decreasing BP, it may be common among those who expose to a high risk of adverse health outcomes, such as heart failure, undernutrition, orthostatic hypertension, and frailty, thus the associations between decreasing BP and mortality may attribute to reverse causality (37). However, some existing studies also supported that BP decline and dramatic BP variation were associated with a higher risk of mortality (38–40). In our study, we found that excess BP trajectories with decreasing trends may associate with a higher risk of mortality, which could be reasonable. A previous study using CLHLS dataset has found a significant increase in hypertension prevalence among the Chinese oldest-old from 1998 to 2014. The mean SBP level decreased from 148.4 ± 24.4 mmHg in 1998 to 130.8 ± 18.7 mmHg in 2005, and then increased to 139.7 ± 22.0 mmHg in 2014 (41). For some old participants, lasting excess BP at relative high level could be possible. Meanwhile, during the aging period, comorbidity, frailty, and other health conditions may decrease BP level. Along with our result that BP began to decline six years prior to death, we believe that it could be reasonable in identifying the BP trajectory with the combination of excess BP and BP decline among the very old people in China. More further studies are needed to explore whether BP variations enhanced or diminish the associations between long-term excess BP and mortality.

It has been well demonstrated that both very low and very high BP is associated with a higher risk of CVD events (41, 42). A *post hoc* observational analysis of a multicenter trial involving 20,330 patients (age >50) found that compared with high-normal (130–<140 mm Hg) SBP, SBP levels during follow-up in the very normal-low (<120 mm Hg), high (140-<150 mm Hg), or very high (≥150 mm Hg) range were associated with 29% (HR = 1.29, 95%CI: 1.07–1.56), 23% (HR = 1.23; 95% CI: 1.07–1.41), 108% (HR = 2.08; 95% CI: 1.83–2.37) increased risk of the first occurrence of stroke (42). According to experience, it is difficult to increase BP among the very old elderly, therefore, our findings may suggest the benefit of long-term stable BP although at a normal high level.

Baseline BP was one of the most important factors that should be taken into consideration in CVD prevention (43). We found that the associations between BP trajectories and all-cause mortality were robust regardless of the baseline BP, while baseline BP may be an important factor when considering the associations between long-term BP trajectories and CVD mortality. A previous multicenter registry study in China found that having a baseline SBP ≤110 mmHg or DBP <70 mmHg was associated with a significantly higher risk of all-cause mortality in patients with AF (44). Future studies are needed to testify the role of baseline BP played in the associations between long-term BP trajectories and mortality.

### 4.2. Terminal BP decline among the Chinese elderly

The terminal decline was typical among the elderly and may somewhat explain the negative association between decreasing trajectory and death among the elderly (45). Our results were similar to a prospective cohort study using data from UK electronic health records (including 144,403 participants older than 80 years old), which found a terminal decline of SBP in the final two years of life (46). However, the variation of BP decline was milder among the Chinese elderly. The possible reason may be differences in meditation strategies, diet patterns, and race between the Chinese and British. Previous studies could better explain the different trajectory patterns of SBP and DBP before death (9). It has been demonstrated that with increasing age, PP and SBP become stronger predictors of coronary heart disease than DBP (47). Meanwhile, previous studies showed that SBP and PP increased in the early old and then decreased in later life, but DBP and MAP showed stabilized declines after 60 years old (10), which were consistent with the patterns of terminal BP trajectories in our study. Terminal BP decline may have potential implications for disease monitoring, risk estimation, and trail design.

Although the potential mechanisms of terminal BP decline have not been well explored, they could be partly explained by the following reasons. First, terminal BP decline may result from cardiovascular and neurological comorbidities, weight loss, dehydration, and polypharmacy and may be a marker of poor health for the elderly. Long-term excess BP and large variation among the elderly may impair endothelial function, lead to inadequate tissue perfusion, and damage cardiac, vascular, tissues, and organs (48, 49). For cardiovascular morbidity such as heart failure, left ventricle systolic dysfunction was associated with BP decreasing (50). Second, excessive antihypertension medications during later life may exist among the Chinese elderly, which may lead to a terminal BP decline. Last but not least, excess mortality of hypertensive left survivors with relatively lower BP.

### 4.3. Strengths and limitations

Our study had several strengths. This analysis was based on a high-quality and nationwide representative sample of the oldest old people in China, which guaranteed the robustness of the results. Furthermore, we used BP trajectory other than a single BP measurement to capture better dynamic changing patterns of BP, which could provide additional values to long-term BP monitoring among the elderly.

However, several limitations of our study merit mention. First, the study showed that long-term excess BP trajectory with decreasing trend was associated with a higher risk of all-cause and CVD mortality, but whether BP variations enhanced or diminish the associations between long-term excess BP and mortality remains unclear. Second, because of data unavailability, we could not adjust for other potential confounders such as antihypertensive meditation and biochemistry indicators (such as total cholesterol, high-density lipoprotein, low-density lipoprotein, et al.). Missing information on anti-hypertension meditation may overestimate the association between BP trajectory and mortality. Third, the unavailability of treatment for hypertension and/or other comorbidities such as diabetes, and dementia made it difficult to explore the potential role of anti-hypertension treatment in the associations between BP trajectories and all-cause mortality. Further studies are needed to address this gap. Forth, CVD mortality in CLHLS was underestimated because of the data collection methods (51), which might underestimate the association between BP trajectories and CVD mortality. And data on the specific type of CVD mortality was unavailable, so the associations between BP trajectories and types of CVD mortality remain unclear. Further studies also need to explore CVD-specific mortality by age and sex and identify the role of age and sex played in the association between BP trajectories and CVD-specific mortality. Fifth, selection bias may exist because we only included participants with at least three BP measurements. People with low adherence, such as frail people, were excluded, which may obscure the true relationship between BP trajectories and mortality. But our results were robust when excluded bedridden participants in the sensitivity analysis. What's more, recall bias and wrong proxy answers might exist in reporting death time and some confounders. And some potential confounders that affect the associations between BP trajectories and all-cause mortality may not be adjusted in the model based on the current literature.

## 5. Conclusion

In the nationwide prospective cohort study, excess BP trajectories with decreasing trend (including SBP, DBP, PP, and MAP), which decreased from an extremely high level and constantly exceeded the normal range, were all found to be associated with a higher risk of all-cause mortality and CVD mortality when compared with stable at normal high-level trajectories. Moreover, terminal SBP/PP trajectories began to decline slightly six years before death, while DBP/MAP showed stabilized decline among Chinese elderly who died older than 60. According to experience, it is difficult to increase BP among the very old elder, therefore, our findings may suggest the benefit of long-term stable BP although at a normal high level. Timely and suitable BP control management among the elderly is urgently needed. And random clinical trials considering frail status, meditation, terminal BP decline ect, are needed to testify these results further and promote optimal BP management strategies for the elderly.

## Data Availability

The datasets analysed for this study can be found in the Peking University Open Research Data Platform (https://opendata.pku.edu.cn/dataset.xhtml?persistentId=doi:10.18170/DVN/WBO7LK).
