# Supplementary material for: Associations between long-term blood pressure trajectory and all-cause and CVD mortality among old people in China
Source: Front Cardiovasc Med. 2023 Aug 17;10:1157327. doi: 10.3389/fcvm.2023.1157327 (PMC10471127; doi:10.3389/fcvm.2023.1157327)
Supplement: Supplementary file 1 [file Table1.pdf]

Supplementary Material

Associations between long-term blood pressure trajectory and all-cause and CVD mortality among old people in China

Huimeng Liu[1,2], Yutong Wang[,2,3,4], Binyan Zhang[2,3,4], Jingchun Liu[2,3,4], Yating Huo[2,3,4], Suixia Cao[2,3,4], Shaowei Wu[1], Yong Wan[5], Xinming Xie[6], Lingxia, Zeng[2,3,4], Hong Yan[2,3,4], Shaonong Dang[2,3,4], Baibing Mi[2,3,4,5,6*]

[1]Department of Occupational and Environmental Health, School of Public Health, Xi'an Jiaotong University Health Science Center, Xi'an, Shaanxi, China.

2 Key Laboratory for Disease Prevention and Control and Health Promotion of Shaanxi Province， Xi'an，Shaanxi, China

[3]Global Health Institute, School of Public Health, Xi'an Jiaotong University Health Science Center, Xi'an, Shaanxi, China

[4]Department of Epidemiology and Biostatistics, School of Public Health, Xi'an Jiaotong University Health Science Center, Xi'an, Shaanxi, China.

[5]Department of Geriatric Surgery, First Affiliated Hospital, Xi'an Jiaotong University, Xi'an, China

[6]Department of Respiratory and Critical Care Medicine, First Affiliated Hospital of Xi'an Jiaotong University, Xi'an, Shaanxi, China

*** Correspondence: Baibing MI**: xjtu.mi@xjtu.edu.cn

1. Supplementary Method

Supplementary Method S1. A brief overview of latent class trajectory analysis (LCTA) and BP trajectory model fitting procedure

Group-based trajectory models are designed to identify clusters of individuals following similar progressions of some behavior or outcome over age(1). Although each individual has a unique development trend of BP, the heterogeneity or distribution of individual variation within the data can be summarized by a finite set of unique polynomial functions. LCTA is mostly used to determine the number of different subgroups of development trends and to fit the development trajectory of each subgroup(2, 3). In the current study, BP is continuous data that is approximately normally distributed, so we assumed a censored normal model.

Suppose $Y_i = \{y_{i1}\ y_{i2}\ y_{i3}, \dots, y_{iT}\}$ represent longitudinal repeated BP measurements of individual *i* over *T* periods. The probability of observing the data trajectory Y_i given membership in group j from risk factor (Z) is:

$$\Pr\left(Y_{i=y_i} \middle| Z_{i=z_i}\right) = \prod_{y_{it=min}} \emptyset \left[\frac{Min-\mu_{ijt}}{\sigma}\right] \prod_{Min<y_{it}<Max} \frac{1}{\sigma} \emptyset \left[\frac{y_{it}-\mu_{ijt}}{\sigma}\right] \prod_{y_{it=Max}} \left[1 - \emptyset \left[\frac{Max-\mu_{ijt}}{\sigma}\right]\right],$$

Where $\mu_{ijt} = \beta_{0j} + age_{it}\beta_{1j} + age_{it}^2\beta_{2j} + \cdots + \varepsilon_{it}\mathrm{\mu_{ijt}} = \mathrm{\beta_{0j}} + \mathrm{age_{it}\beta_{1j}} + \mathrm{age_{it}^2\beta_{2j}} + \cdots + \mathrm{\varepsilon_{it}}$

ε_{it} is assumed to be normally distributed with a zero mean and a constant standard deviation σ.

Following the current guideline of PROC TRAJ in SAS, the model fitting procedure was listed as follows (1):

Step 1. Select a plausible provisional **s**cope model with several groups. Based on previous studies and plausible clinical patterns, we initially constructed a scoping model with two to five trajectory groups of long-term BP change trends among the elderly in China(4)**.**

Step 2. Refine the model from Step 1 to confirm the optimal trajectory number. The fitted number was chosen based on the lowest Bayesian information criterion (BIC)(5). Besides, the value of group membership probability should be equal to or greater than 5% to ensure a sufficient sample size in each group(6). For SBP trajectory fitting, BIC was the lowest when the group number was 3. (**Supplementary Table S1**).

Step 3. Refine optimal model structure by testing the best-fitting shape. We used a stepwise approach in establishing polynomial order, with all groups initially set to cubic order. Then change the trajectory to quadratic, linear, and intercept according to BIC and the significance of the parameters of each model. It should be noted that the Proc Traj allows the estimation of up to a fourth-order polynomial(7).

Step 4. Perform model assessments. We calculated the posterior probability of being assigned to each trajectory class for each participant. An average posterior probability above 70% is acceptable(8). In our study, the average posterior probability for all BP trajectories was higher than 75% (**Supplementary Table S2)**. BIC values may decrease in some situations as more groups are added (such as MAP trajectory fitting). Therefore, the BIC value might not always provide the optimum selection criteria, and the model was selected by balancing all criteria and considering model parsimony, adequacy, and practical implications(1).

Supplementary Method S2. Detailed information on the frail index (FI) calculation

In this study, 35 health deficits were included in FI calculating using the cumulative deficits method base on the standard procedure(9). 35 health deficits included self-rated health, psychological characteristics, activities of daily living (ADL), instrumental activities of daily living (IADL), hearing or vision impairment, cognitive functioning, and chronic diseases. Each item was dichotomous or ordinal, recorded from 0 to 1, and closer to 1 meant more severe health deficits(10) (specific items and scoring rules were in **Supplementary Table S3**). Finally, FI was calculated as the total score of the health deficits divided by the total possible number of deficits. Missing data on the health deficits were not included in both denominator and the numerator(11). Furthermore, frailty was categorized into non-frail for those with FI⩽0.1, pre-frail for 0.1<FI⩽0.21, and frail for FI>0.21(12).

Supplementary Method S3. Identify BP trajectories 15 years prior to death by a mixed-effects model

Because of the limited data on BP measurements more than 15 years before death, we only included those who died within 15 years after enrollment in the study. Finally, 6,010 decedents were included in this analysis. We tested three mixed models with linear, quadratic, and cubic items to test the non-linear relationship between the time before death and measured BP. Model 1 is a linear mixed effect model with random intercept constructed using the formula provided below (take SBP as an example).

$$SBP_{ij} = \beta_0 + \beta_1 t_{ij} + \beta_2 age_{ij} + \beta_3 gender_{ij} + \beta_4 wave_{ij} + b_{0i} + e_{ij},$$

$$i = 1,2,\ldots,n, j = 1,2,\ldots,15$$

(Model 1: Linear model)

Where SBP_{ij} represents the estimated SBP in mmHg for individual i at time j (in the year, ranging from 1-15 years before death), β_0 represents the intercept (estimated SBP at death), $\beta_1$represents expected SBP change from j years before death to death, β_2-β_4 are regression coefficients, b_{0i} represents the random effect for individual i, and e_{ij} is the error term, which is assumed to be independent, identical, and normally distributed with a mean of zero and constant variance.

Model 2 is a mixed-effects model to predict expected SBP by 1-year distance to death with the quadratic specification of the time to death:

$$SBP_{ij} = \beta_0 + \beta_{11} t_{ij} + \beta_{12} t_{ij}^2 + \beta_2 age_{ij} + \beta_3 gender_{ij} + \beta_4 age_{ij} + \beta_4 wave_{ij} + b_{0i} + b_{1i} t_{ij} + e_{ij},$$

$$i = 1,2,\ldots,n, j = 1,2,\ldots,15$$

(Model 2: Quadratic model)

Model 3 is a mix-effects model to predict expected SBP by 1-year distance to death with the cubic specification of the time to death:

$$SBP_{ij} = \beta_0 + \beta_{11}t_{ij} + \beta_{12}t_{ij}^2 + \beta_{13}t_{ij}^3 + \beta_2 age_{ij} + \beta_3 gender_{ij} + \beta_4 age_{ij} + \beta_4 wave_{ij} + b_{0i} + b_{1i}t_{ij} + e_{ij},$$

$$i = 1,2,\dots,n, j = 1,2,\dots,15$$

(Model 3: Cubic model)

As shown in **Supplementary Table S4**, only 0.69% BP measurements of the decedents were conducted more than 15 years before death, so we only included those who died within 15 years of enrollment.

The best-fitted model was selected based on the lowest BIC (**Supplementary Table S5**). The mean BP (SBP, DBP, PP, MAP) of each year prior to death was calculated and plot the BP trajectories. Quadratic models were the fittest for both SBP, DBP and PP. For MAP, the linear model was the fittest.

Supplementary Method S4. Detailed information on Multiple imputations (MI)

MI is an iterative process based on a Bayesian approach that alternates between estimating the parameters for this distribution and the imputed values predicted by randomly obtained from its predictive distribution[1362]. Under the assumption of missing at random (MAR), we generated five completed datasets and pooled the imputed data sets according to Rubin's rules[13]. As suggested in the literature[14], we included all the relevant variables likely to be used in the subsequent analyses. We chose to use the MI method for several reasons: (1) the missing proportions of confounders were not too large (<40%), (2) the missing were not at random (MNAR), and the missing completely at random (MCAR) assumption seemed implausible for our dataset, and (3) missing values on a confounder were likely to be influenced by the exposure, outcome, and other confounders[15]. A missing portion of the data was shown in **Supplementary Table S6.** Miss values of confounders range from 0% to 12.36%, which may be due to recall difficulties. The largest missing portion was on ethnicity. And the missing portion of other covariates was less than 1%, which was small enough to be ignored[15]. So, the primary analyses of our study were reliable, and analyses based on imputed datasets were recognized as sensitivity analyses to test the robustness of the results.

2. Supplementary Figures and Tables

2.1 Supplementary Figures

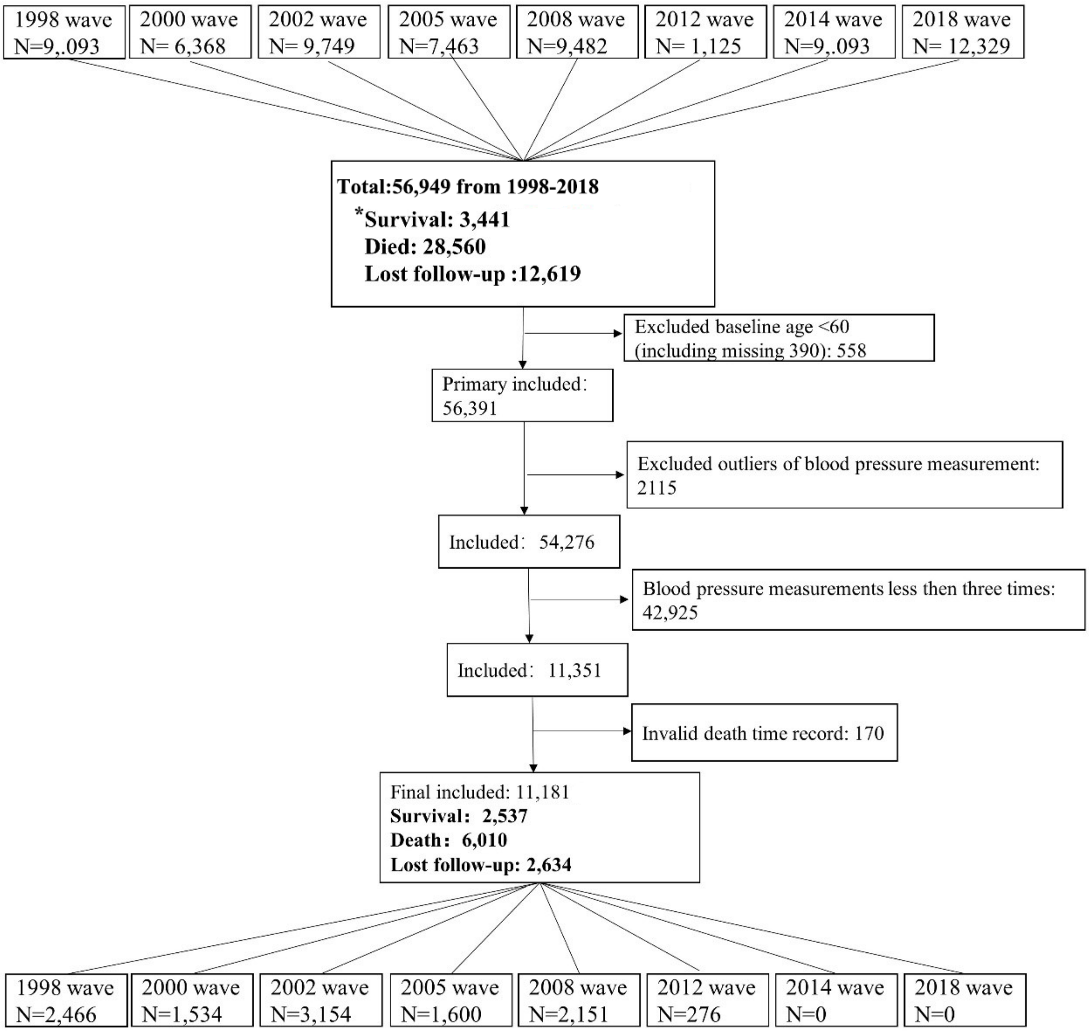

*The 12,319 participants involved in the 2018 cross-sectional survey were not included in follow-up survival status statistician.

Supplementary Figure S1. Study sample flow chart

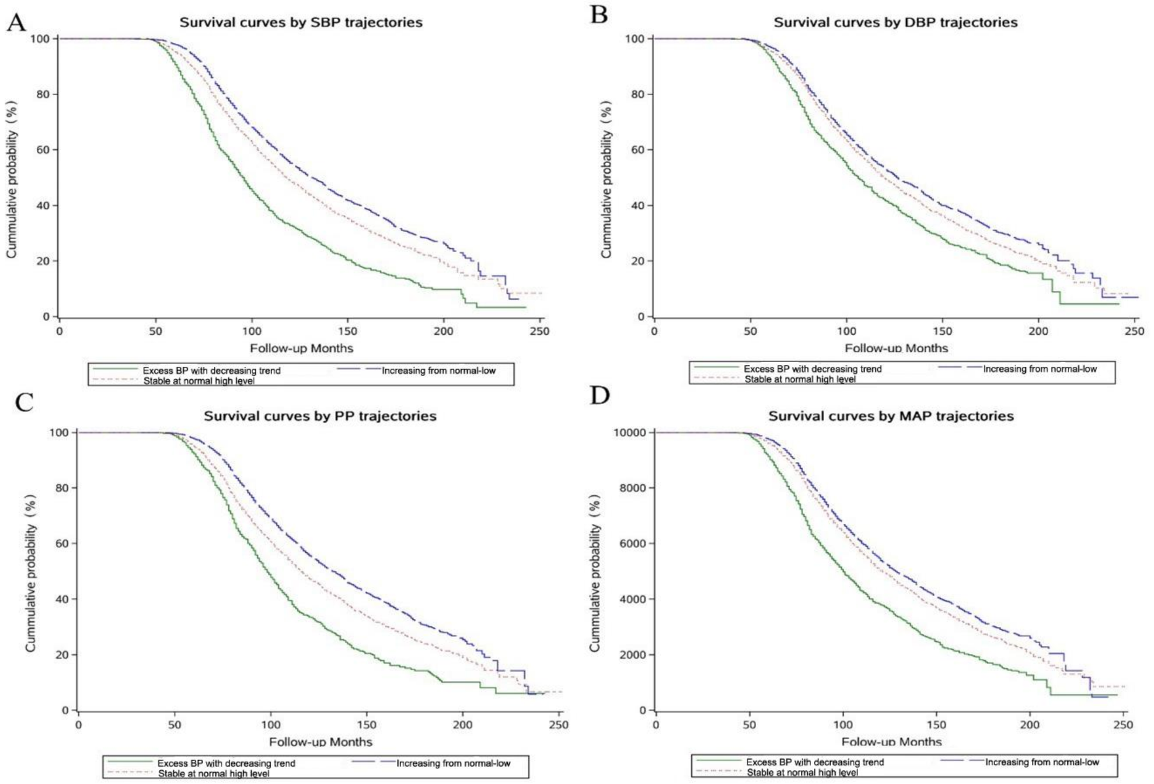

*Cox -proportional hazard model was used to plot the survival curves.

†A Systolic blood pressure (SBP), B Diastolic blood pressure (DBP), C Pulse pressure (PP), D Mean arterial pressure (MAP)

Supplementary Figure S2. Survival curve by BP trajectories

A

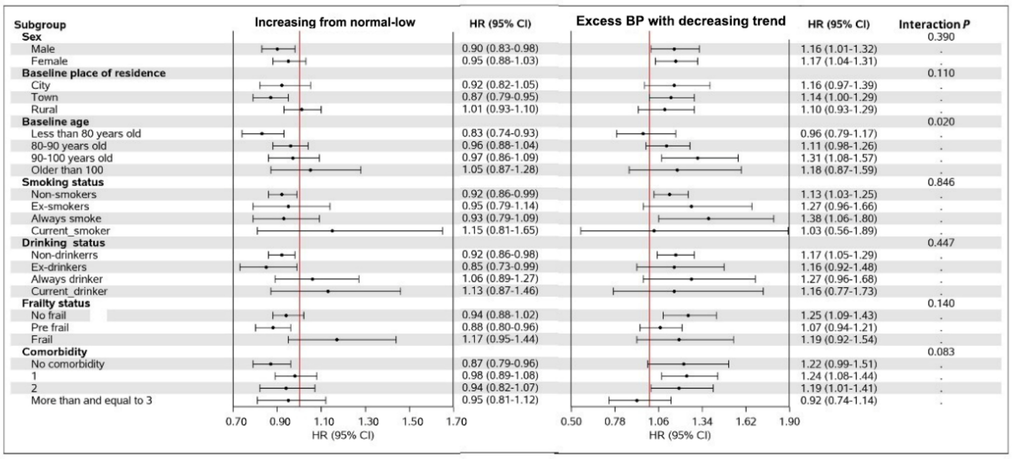

* Reference group was "stable at normal high level" group

B

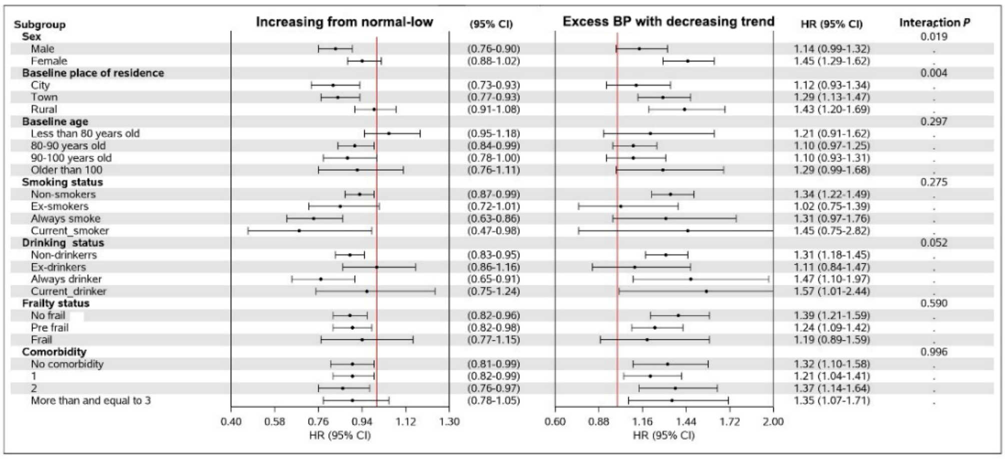

* Reference group was "stable at normal high level" group

C

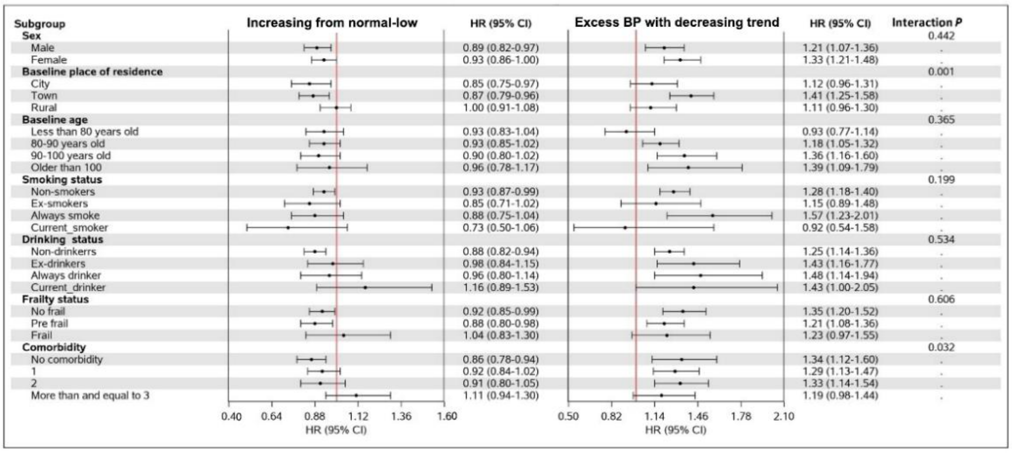

* Reference group was "stable at normal high level" group

Supplementary Figure S3. Forest plot of the association with DBP (A), PP (B), MAP (C) trajectories and mortality in different subgroups

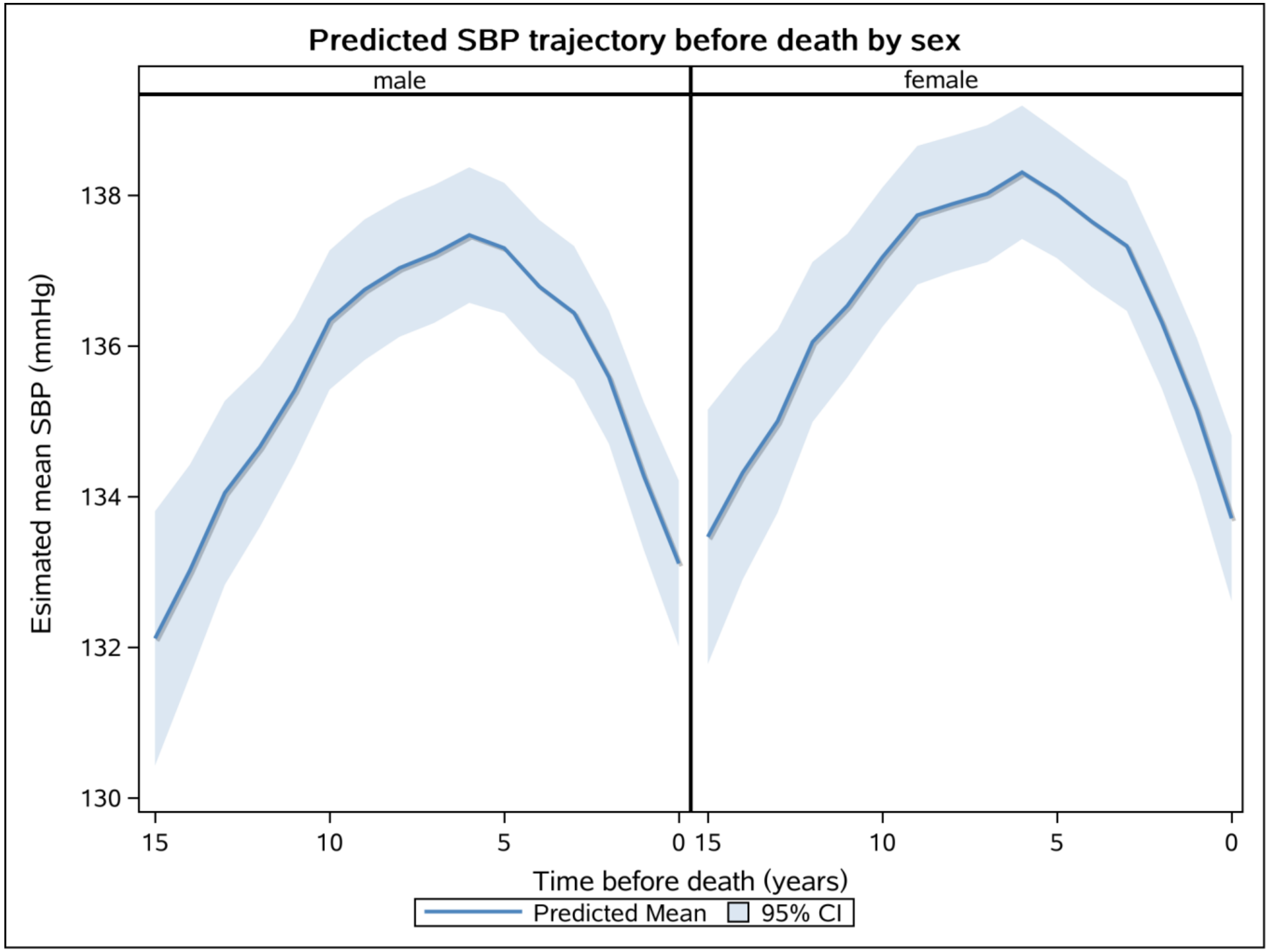

Supplementary Figure S4. Estimated mean SBP in the 15 years before death by sex

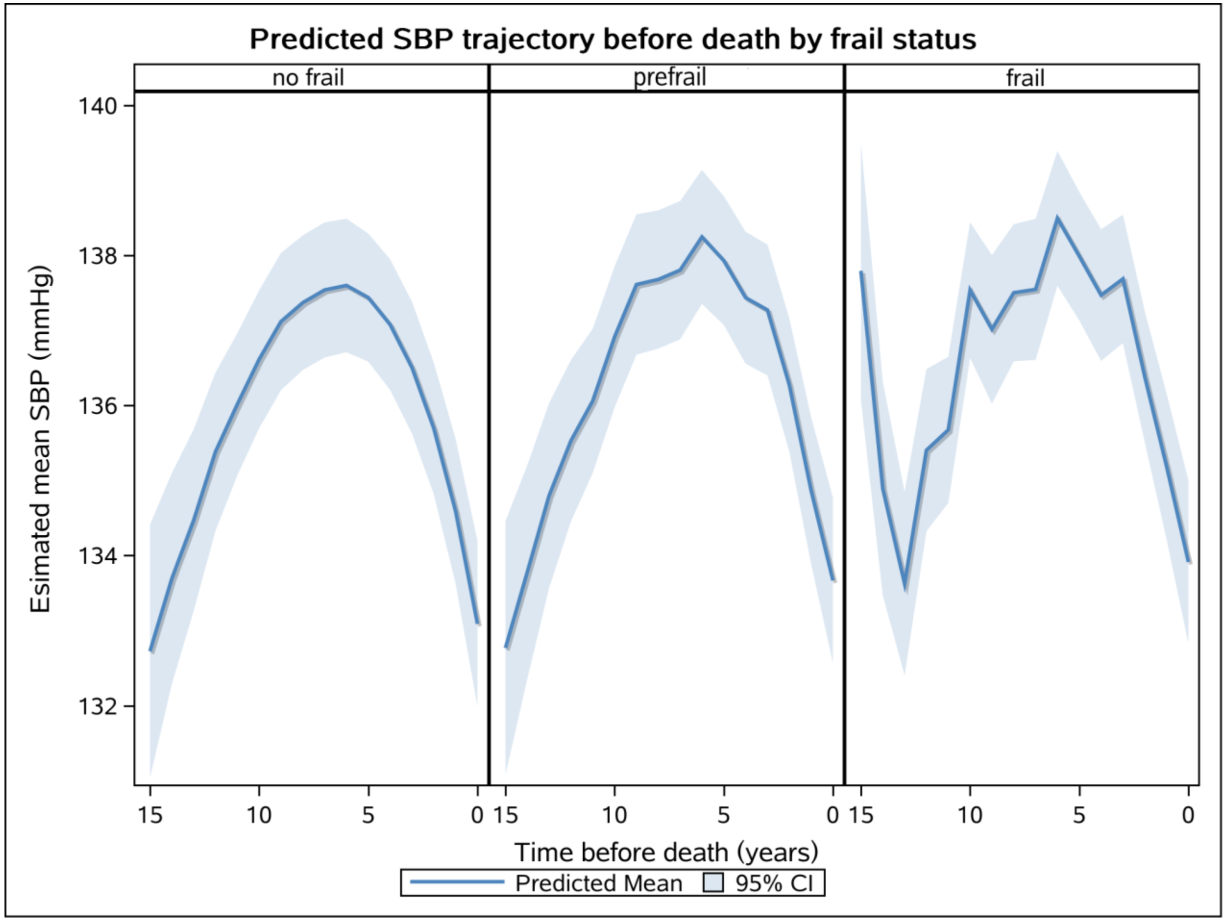

Supplementary Figure S5. Estimated mean SBP in the 15 years before death by frail status

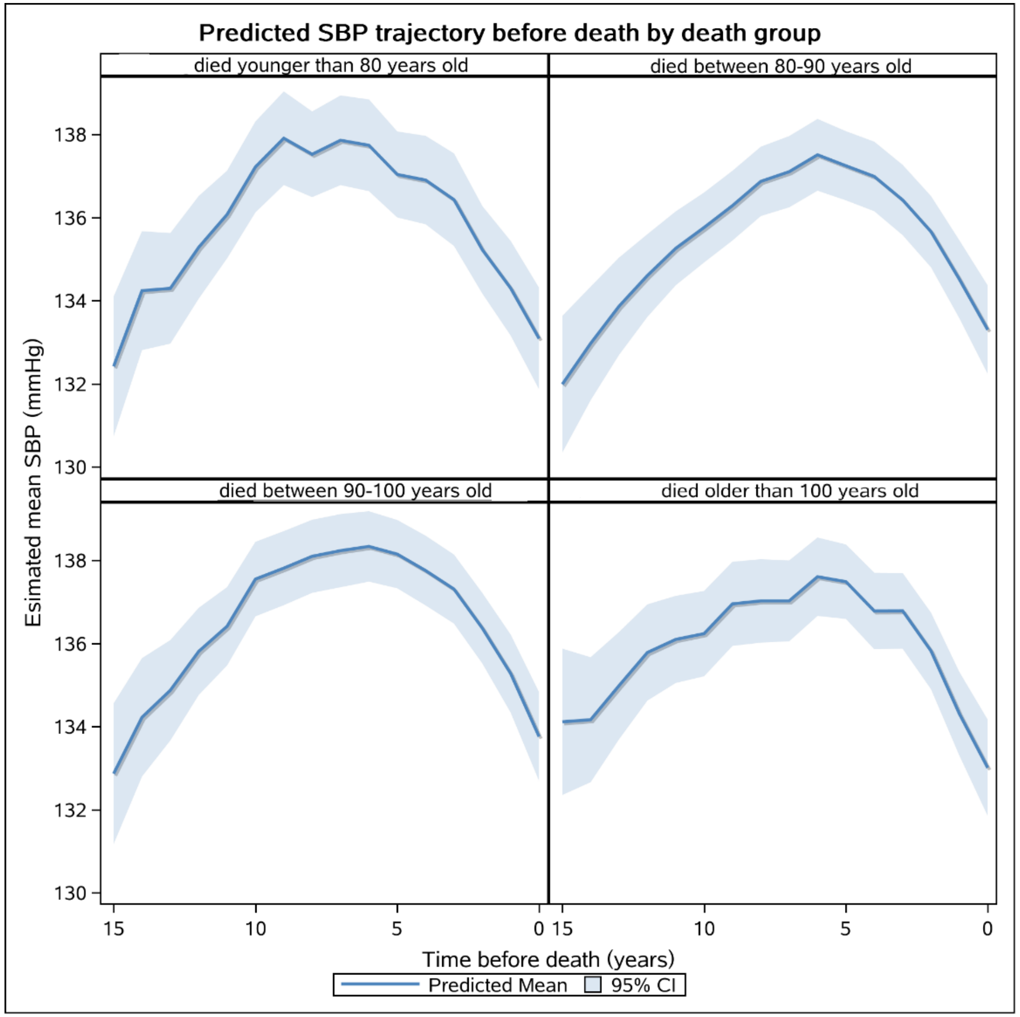

Supplementary Figure S**6**. Estimated mean SBP in the 15 years before death by death groups

2.2 Supplementary Tables

Supplementary Table S1. Number of selected SBP trajectories based on the Bayesian Information Criterion (BIC)

**Number of Group**	**BIC**	**Group membership probability (%)**				
		Group1	Group2	Group3	Group4	Group5
			SBP			
2	-187397.2636	68.87	31.13			
3	-186711.1129	46.56	41.91	11.53		
4	-186739.0789	0.00	41.91	46.56	11.53	
5	-186479.1330	0.00	45.31	21.95	27.52	5.22
			DBP			
2	-166294.819	85.48	14.52			
3	-166256.916	50.90	38.00	11.10		
4	-166257.544	14.82	77.89	6.37	0.92	
5	-166161.430	1.52	72.41	1.23	23.18	1.67
			PP			
2	-184163.1961	89.04	10.96			
3	-184137.2899	44.34	44.71	10.96		
4	-184087.5529	1.48	8.74	10.06	79.72	
5	-184110.5188	1.44	9.89	21.75	58.16	8.76
			MAP			
2	-166919.5416	69.31	30.69			
3	-166206.2029	50.27	35.08	14.65		
4	-166163.7365	48.60	0.84	35.97	14.59	
5	-165950.3687	22.69	46.02	24.30	0.80	6.19

*Abbreviation: BIC Bayesian Information Criterion, SBP Systolic Blood Pressure, DBP Diastolic Blood Pressure, PP Pulse Pressure, MAP Mean Arterial Pressure

Supplementary Table S2. Parameters estimated for BP trajectory

	Parameter Estimate (SE)*					
**BP trajectory change pattern**	**Intercept term**	**Linear term**	**Quadratic term**	**Cubic term**	**Group membership probability (%)**	**Average posterior probability (%)**
			SBP			
Group 1	152.243 (19.561)	0.658 (0.455)	-0.007 (0.003)		46.43	84.91
Group 2	-378.813 (61.075)	16.943 (2.179)	-0.187 (0.026)	0.001 (1×10-3)	42.23	87.58
Group 3	103.261 (9.341)	0.997 (0.221)	-0.007 (0.001)		11.34	87.13
			DBP			
Group 1	297.321 (35.252)	-7.439 (1.262)	0.086 (0.015)	3×10-4 (5×10-5)	50.94	84.92
Group 2	-133.834 (38.246)	7.345 (1.363)	-0.085 (0.016)	3×10-4 (6×10-5)	37.99	89.04
Group 3	636.576 (85.655)	-18.379 (3.037)	0.206 (0.036)	7×10-4 (1×10-3)	11.07	85.22
			PP			
Group 1	95.400 (16.516)	-1.601 (0.603)	0.023 (0.007)	1×10-4 (3×10-5)	44.34	75.94
Group 2	-525.662 (50.444)	19.278 (1.800)	-0.213 (0.021)	8×10-4 (8×10-5)	44.71	94.07
Group 3	48.431 (18.246)	1.048 (0.424)	-0.009 (0.002)		10.96	80.21
			MAP			
Group 1	190.240 (38.048)	-3.272 (1.361)	0.040 (0.016)	2×10-4 (6×10-5)	50.27	85.69
Group 2	-200.847 (42.202)	10.075 (1.506)	-0.114 (0.018)	4×10-4 (7×10-5)	35.08	87.39
Group 3	450.696 (74.117)	-11.490 (2.637)	0.131 (0.031)	5×10-4 (1×10-4)	14.65	87.79

*Parameter estimate presented the shape of each pattern of trajectory over time. The intercept term is interpreted as the expected level of BP at the first measurement. Linear, quadratic, and cubic terms are interpreted as the linear, quadratic, and cubic slope of BP by age.

†Abbreviation: BP Blood Pressure, SE Standard Error.

Supplementary Table S3. 35 health deficits included in the frailty index

No.	Variables	Values
1	Self-reported health	very good=0; good=0.25; average=0.5; bad=0.75; very bad=1
2	Feel fearful or anxious	always=1; often=0.75; sometimes=0.5; seldom=0.25; never=0
3	Feel useless because of age	always=1; often=0.75; sometimes=0.5; seldom=0.25; never=0
4	Look on the bright side of things	always=0; often=0.25; sometimes=0.5; seldom=0.75; never=1
5	Keep my belongings neat and clean	always=0; often=0.25; sometimes=0.5; seldom=0.75; never=1
6	Make own decisions	always=0; often=0.25; sometimes=0.5; seldom=0.75; never=1
7	Bathing	without assistance=0; assistance with 1 body part=0.5; assistance with >1 body part=1
8	Dressing	without assistance=0; some assistance=0.5; unable to dress without assistance=1
9	Use of toilet	without assistance=0; some assistance=0.5; unable to use toilet without assistance=1
10	Indoor activities	without assistance=0; some assistance=0.5; a lot of assistance=1
11	Continence	able=0; occasional incontinence=0.5; frequent incontinence=
12	Feeding	without assistance=0; some assistance=0.5; a lot of assistance=1
13	Housework at present	almost every day=0; not daily, but at least once a week=0.5; not weekly, but at least once a month=0.5; not monthly, but sometimes=0.5; never=1
14	Visual function	can see and distinguish the break in the circle=0; can see but not distinguish the break in the circle=0.33; cannot see=0.67; blind=1
15	Hearing ability	can hear without a hearing aid=0; can hear with a hearing aid=0.33; partly deaf, despite using a hearing aid=0.67; deaf=1
16	Cardiac rhythm	regular=0; irregular=1
17	Hand behind neck	right hand=0.5; left hand=0.5; both hands=0; neither hand=1
18	Hand behind lower back	right hand=0.5; left hand=0.5; both hands=0; neither hand=1
19	Able to stand up from sitting	yes, without using hands=0; yes, using hands=0.5; no=1
20	Able to pick up a book from the floor	yes, standing=0; yes, sitting=0.5; no=1

No.	Variables	Values
21	Able to use chopsticks to eat	yes=0; no=1
22	Number of steps used to turn around a 360-degree turn without help	≤4=0; >4=0.5; cannot turn around=1
23	Number of times suffering from serious illness in the past two years	0=0; 1=1; >1=2
24	Hypertension	yes=1; no=0
25	Diabetes	yes=1; no=0
26	Heart disease	yes=1; no=0
27	Stroke or cerebrovascular disease	yes=1; no=0
28	Bronchitis, emphysema, pneumonia, asthma	yes=1; no=0
29	Tuberculosis	yes=1; no=0
30	Cancer	yes=1; no=0
31	Gastric or duodenal ulcer	yes=1; no=0
32	Parkinson’s disease	yes=1; no=0
33	Bedsores	yes=1; no=0
34	Dementia	yes=1; no=0
35	Interviewer rated-health	surprisingly healthy=0; relatively healthy=0.33; moderately unhealthy=0.67; very unhealthy=1

Supplementary Table S4. Number of BP mearements at different death year after enrollment

Year before death	Number (%)
0	792 (3.73)
1	2061 (9.70)
2	2149 (10.11)
3	2140 (10.07)
4	2342 (11.02)
5	2264 (10.65)
6	2159 (10.16)
7	1974 (9.29)
8	1510 (7.10)
9	1193 (5.61)
10	803 (3.78)
11	575 (2.71)
12	497 (2.34)
13	300 (1.41)
14	201 (0.95)
15	146 (0.69)
16	95 (0.45)
17	35 (0.16)
18	11 (0.05)
19	6 (0.03)
20	1 (0.00)

Supplementary Table S5. Mixed-effects model selection of BP trajectories 15 years before death

Tested Model	BIC	AIC	-2 Res Log Likelihood
		SBP	
Model 1	186872.2	186858.8	186854.8
**Model 2**	**186823.0**	**186809.6**	**186805.6**
Model 3	186830.5	186817.1	186813.1
		DBP	
Model 1	164071.8	164058.4	164054.4
**Model 2**	**164067.8**	**164054.4**	**164050.4**
Model 3	164079.0	164065.6	164061.6
		PP	
Model 1	182207.3	1821993.9	182189.9
**Model 2**	**182108.7**	**182095.3**	**182091.3**
Model 3	182115.1	182101..77	182097.7
		MAP	
**Model 1**	**166512.8**	**166499.4**	**1664955.4**
Model 2	166518.4	166505.0	166501.0
Model 3	166528.9	166515.5	166511.5

*Model 1: Linear model; Model 2: Quadratic model; Model 3: Cubic model.

†Best fitted model was highlighted in bolded.

‡Abbreviation: AIC Akaike Information Criterion, BIC Bayesian Information Criterion, BP Blood Pressure, SBP Systolic Blood Pressure, DBP Diastolic Blood Pressure, PP Pulse Pressure, MAP Mean Arterial Pressure,

Supplementary Table S6. The missing portion of adjusted covariates

Variable	Number of missing (%)
Sex	0 (0.00)
Ethnicity	1382 (12.36)
Occupation before retirement	33 (0.30)
Baseline marriage status	5 (0.00)
Place of residence at baseline	0 (0.00)
Average household income	2 (0.00)
Baseline regular exercise	3 (0.00)
Smoking status	19 (0.17)
Drinking status	26 (0.23)
Number of comorbidities	0 (0.00)
Frail status	0 (0.00)

Supplementary Table S7. Demographic characteristics of study participants by DBP trajectories

Characteristics	Stable at normal high level	Increasing from normal-low	Excess BP trajectory with decreasing trend	*P* value*
Number of participants (%)	6027 (53.90)	4070 (36.40)	1084 (9.70)	
BP measurements n (%)	23152 (54.00)	15667 (36.54)	4052 (9.45)	
Mean baseline age (SD)	80.74 (10.66)	80.73 (10.83)	82.39 (10.39)	<0.001
Sex n (%)				
Male	2764 (45.86)	1865 (45.82)	491 (45.30)	0.941
Female	3263 (54.14)	2205 (54.18)	593 (54.70)	
Ethnicity n (%)				
Han nationality	5035 (93.97)	3176 (91.87)	924 (93.90)	<0.001
Others	323 (6.03)	281 (8.13)	60 (6.10)	
Average household income (yuan) n (%)				
Less than 5,000	1873 (31.08)	1095 (26.91)	409 (37.73)	
5,000-19,999	1865 (30.95)	1328 (32.64)	308 (28.41)	<0.001
More than 20,000	2288 (37.97)	1646 (40.45)	367 (33.86)	
Baseline place of residence n (%)				
City	1388 (23.03)	895 (21.99)	272 (25.09)	
Town	1808 (30.00)	1083 (26.61)	420 (38.75)	<0.001
Rural	2831 (46.97)	2092 (51.40)	392 (36.16)	
Main occupation before retirement n (%)				
Professional and technical personnel	276 (4.59)	199 (4.90)	64 (5.92)	
Governmental, institutional, or managerial personnel	209 (3.48)	151 (3.72)	42 (3.89)	
Agriculture, forest, animal husbandry	1620 (26.97)	1053 (25.94)	331 (30.62)	
Fishery worker	88 (1.46)	57 (1.40)	15 (1.39)	<0.001

**Characteristics**	**Stable at normal high level**	**Increasing from normal-low**	**Excess BP trajectory with decreasing trend**	***P* value***
Industrial worker	2844 (47.34)	1966 (48.42)	420 (38.85)	
Commercial or service worker	474 (7.89)	283 (6.97)	81 (7.49)	
Military personnel	28 (0.47)	26 (0.64)	17 (1.57)	
Housework	367 (6.11)	239 (5.89)	86 (7.96)	
Others	94 (1.56)	77 (1.90)	21 (1.94)	
Marriage at baseline n (%)				
Currently married and living with spouse	2402 (39.87)	1633 (40.14)	390 (36.01)	
Separated	134 (2.22)	90 (2.21)	15 (1.39)	0.118
Divorced	32 (0.53)	19 (0.47)	7 (0.65)	
Widowed	3379 (56.08)	2284 (56.15)	658 (60.76)	
Never married	78 (1.29)	42 (1.03)	13 (1.20)	
Smoking status n (%)				
Non-smokers	4455 (74.04)	2988 (73.52)	804 (74.38)	
Ex-smokers	631 (10.49)	397 (9.77)	119 (11.01)	0.185
Always smoke	719 (11.95)	547 (13.46)	129 (11.93)	
Current smoker	212 (3.52)	132 (3.25)	29 (2.68)	
Drinking status n (%)				
Non-drinkers	4271 (70.99)	2855 (70.32)	780 (72.29)	
Ex-drinkers	786 (13.07)	563 (13.87)	144 (13.35)	
Always drinker	635 (10.56)	425 (10.47)	106 (9.82)	0.757
Current drinker	324 (5.39)	217 (5.34)	49 (4.54)	
Exercise at baseline n (%)				
Yes	2073 (34.40)	1405 (34.53)	386 (35.64)	0.730
No	3953 (65.60)	2664 (65.47)	697 (64.36)	
Frail status n (%)				
No frail	3469 (57.56)	2511 (61.70)	443 (40.87)	
Pre frail	2135 (35.42)	1343 (33.00)	518 (47.79)	<0.001
Frail	423 (7.02)	216 (5.31)	123 (11.35)	
Comorbidity† n (%)				

Characteristics	Stable at normal high level	Increasing from normal-low	Excess BP trajectory with decreasing trend	*P* value*
No comorbidity	1619 (26.86)	1329 (32.65)	141 (13.01)	<0.001
1	1959 (32.50)	1363 (33.49)	381 (35.15)	
2	1357 (22.52)	779 (19.14)	310 (28.60)	
More than and equal to 3	1092 (18.12)	599 (14.72)	252 (23.25)	
CVD death n (%)	175 (2.90)	105 (2.58)	41 (3.78)	<0.001

*ANOVA was used to test variance across BP trajectories for continuous variables and $\chi 2$ test for categorical variables.
†Number of following chronic diseases during the follow-up period: hypertension, diabetes, coronary heart disease, cardiovascular disease, pulmonary diseases, tuberculosis, cancer, tumor, Parkinson.
‡Abbreviation: SD Standard Deviation, DBP Diastolic Blood Pressure, CVD Cardiovascular Disease

Supplementary Table S8. Demographic characteristics of the study participants by PP trajectories

Characteristics	Stable at normal high level	Increasing from normal-low	Excess BP trajectory with decreasing trend	*P* value*
Number of participants (%)	4930 (44.09)	5123 (45.82)	1128 (10.09)	
BP measurements n (%)	3963 (9.24)	20285 (47.31)	18623 (43.44)	
Mean baseline age (SD)	81.45 (10.51)	79.43 (10.79)	85.11 (9.77)	<0.001
Sex n (%)				
Male	2279 (46.23)	2409 (47.02)	432 (38.30)	<0.001
Female	2651 (53.77)	2714 (52.98)	696 (61.70)	
Ethnicity n (%)				
Han nationality	4020 (93.21)	4206 (92.85)	909 (95.08)	0.044
Others	293 (6.79)	324 (7.15)	47 (4.92)	
Average household income (yuan) n (%)				
Less than 5,000	1612 (32.70)	1355 (26.45)	410 (36.35)	
5,000-19,999	1506 (30.55)	1654 (32.29)	341 (30.23)	<0.001
More than 20,000	1811 (36.74)	2113 (41.25)	377 (33.42)	
Baseline place of residence n (%)				
City	1177 (23.87)	1085 (21.18)	293 (25.98)	
Town	1602 (32.49)	1308 (25.53)	401 (35.55)	<0.001
Rural	2151 (43.63)	2730 (53.29)	434 (38.48)	
Main occupation before retirement n (%)				
Professional and technical personnel	234 (4.76)	252 (4.93)	53 (4.70)	
Governmental, institutional, or managerial personnel	189 (3.85)	182 (3.56)	31 (2.75)	
Agriculture, forest, animal husbandry	1433 (29.17)	1215 (23.78)	356 (31.59)	<0.001
Fishery worker	74 (1.51)	79 (1.55)	7 (0.62)	
Industrial worker	2164 (44.06)	2599 (50.87)	467 (41.44)	
Commercial or service worker	353 (7.19)	393 (7.69)	92 (8.16)	
Military personnel	32 (0.65)	36 (0.70)	3 (0.27)	

Characteristics	Stable at normal high level	Increasing from normal-low	Excess BP trajectory with decreasing trend	*P* value*
Housework	343 (6.98)	247 (4.83)	102 (9.05)	
Others	81 (1.65)	95 (1.86)	16 (1.42)	
Marriage at baseline n (%)				
Currently married and living with spouse	1903 (38.62)	2147 (41.93)	375 (33.24)	<0.001
Separated	97 (1.97)	125 (2.44)	17 (1.51)	
Divorced	20 (0.41)	31 (0.61)	7 (0.62)	
Widowed	2848 (57.80)	2757 (53.84)	716 (63.48)	
Never married	59 (1.20)	61 (1.19)	13 (1.15)	
Smoking status n (%)				
Non-smokers	3645 (74.10)	3704 (72.37)	898 (79.82)	<0.001
Ex-smokers	513 (10.43)	537 (10.49)	97 (8.62)	
Always smoke	613 (12.46)	675 (13.19)	107 (9.51)	
Current smoker	148 (3.01)	202 (3.95)	23 (2.04)	
Drinking status n (%)				
Non-drinkers	3500 (71.18)	3557 (69.57)	849 (75.47)	0.011
Ex-drinkers	656 (13.34)	713 (13.94)	124 (11.02)	
Always drinker	510 (10.37)	559 (10.93)	97 (8.62)	
Current drinker	251 (5.10)	284 (5.55)	55 (4.89)	
Exercise at baseline n (%)				
Yes	1669 (33.87)	1814 (35.42)	381 (33.78)	0.222
No	3259 (66.13)	3308 (64.58)	747 (66.22)	
Frail status n (%)				
No frail	2749 (55.76)	3176 (61.99)	498 (44.15)	<0.001
Pre frail	1823 (36.98)	1650 (32.21)	523 (46.37)	
Frail	358 (7.26)	297 (5.80)	107 (9.49)	
Comorbidity† n (%)				
No comorbidity	1349 (27.36)	1523 (29.73)	217 (19.24)	<0.001
1	1624 (32.94)	1691 (33.01)	388 (34.40)	
2	1091 (22.13)	1051 (20.52)	304 (26.95)	
More than and equal to 3	866 (17.57)	858 (16.75)	219 (19.41)	

**Characteristics**	**Stable at normal high level**	**Increasing from normal-low**	**Excess BP trajectory with decreasing trend**	***P* value***
CVD death n (%)	151 （3.06）	117 (2.28)	53 (4.70)	<0.001

*ANOVA was used to test variance across BP trajectories for continuous variables and χ2 test for categorical variables.

† Number of following chronic diseases during the follow-up period: hypertension, diabetes, coronary heart disease, cardiovascular disease, pulmonary diseases, tuberculosis, cancer, tumor, Parkinson

‡ Abbreviation: SD Standard Deviation, PP Pulse Pressure, CVD Cardiovascular Disease.

Supplementary Table S9. Demographic characteristics of the study participants by MAP trajectories

Characteristics	Stable at normal high level	Increasing from normal-low	Excess BP trajectory with decreasing trend	*P* value*
Number of participants (%)	5770 (51.61)	3883 (34.73)	1528 (13.76)	
BP measurements n (%)	5553 (12.95)	15123 (35.27)	22195 (51.77)	
Mean baseline age (SD)	81.18 (10.59)	80.81 (10.93)	83.95 (10.32)	<0.001
Sex n (%)				
Male	2668 (46.24)	1813 (46.69)	639 (41.82)	0.003
Female	3102 (53.76)	2070 (53.31)	889 (58.18)	
Ethnicity n (%)				
Han nationality	4794 (94.20)	3060 (91.51)	1281 (93.78)	<0.001
Others	295 (5.80)	284 (8.49)	85 (6.22)	
Average household income (yuan) n (%)				
Less than 5,000	1731 (30.01)	1055 (27.18)	591 (38.68)	
5,000-19,999	1791 (31.05)	1261 (32.48)	449 (29.38)	<0.001
More than 20,000	2247 (38.95)	1566 (40.34)	488 (31.94)	
Baseline place of residence n (%)				
City	1298 (22.50)	852 (21.94)	405 (26.51)	
Town	1713 (29.69)	988 (25.44)	610 (39.92)	<0.001
Rural	2759 (47.82)	2043 (52.61)	513 (33.57)	
Main occupation before retirement n (%)				
Professional and technical personnel	253 (4.40)	196 (5.06)	90 (5.91)	
Governmental, institutional, or managerial personnel	208 (3.62)	143 (3.69)	51 (3.35)	

**Characteristics**	**Stable at normal high level**	**Increasing from normal-low**	**Excess BP trajectory with decreasing trend**	***P* value***
Agriculture, forest, animal husbandry	1557 (27.06)	968 (24.99)	479 (31.47)	
Fishery worker	86 (1.49)	55 (1.42)	19 (1.25)	<0.001
Industrial worker	2722 (47.31)	1930 (49.83)	578 (37.98)	
Commercial or service worker	458 (7.96)	263 (6.79)	117 (7.69)	
Military personnel	32 (0.56)	23 (0.59)	16 (1.05)	
Housework	346 (6.01)	209 (5.40)	137 (9.00)	
Others	83 (1.44)	79 (2.04)	30 (1.97)	
Marriage at baseline n (%)				
Currently married and living with spouse	2321 (40.24)	1574 (40.56)	530 (34.71)	
Separated	122 (2.12)	94 (2.42)	23 (1.51)	
Divorced	30 (0.52)	19 (0.49)	9 (0.59)	<0.001
Widowed	3221 (55.84)	2156 (55.55)	944 (61.82)	
Never married	74 (1.28)	38 (0.98)	21 (1.38)	
Smoking status n (%)				
Non-smokers	4270 (74.11)	2805 (72.37)	1172 (76.90)	
Ex-smokers	586 (10.17)	412 (10.63)	149 (9.78)	0.027
Always smoke	708 (12.29)	523 (13.49)	164 (10.76)	
Current smoker	198 (3.44)	136 (3.51)	39 (2.56)	
Drinking status n (%)				
Non-drinkers	4077 (70.77)	2710 (69.99)	1119 (73.52)	
Ex-drinkers	765 (13.28)	531 (13.71)	197 (12.94)	0.223
Always drinker	610 (10.59)	421 (10.87)	135 (8.87)	
Current drinker	309 (5.36)	210 (5.42)	71 (4.66)	

Characteristics	Stable at normal high level	Increasing from normal-low	Excess BP trajectory with decreasing trend	*P* value*
Exercise at baseline n (%)				
Yes	1989 (34.48)	1359 (35.01)	516 (33.79)	0.684
No	3780 (65.52)	2523 (64.99)	1011 (66.21)	
Frail status n (%)				
No frail	3334 (57.78)	2475 (63.74)	614 (40.18)	<0.001
Pre frail	2032 (35.22)	1227 (31.60)	737 (48.23)	
Frail	404 (7.00)	181 (4.66)	177 (11.58)	
Comorbidity† n (%)				
No comorbidity	1545 (26.78)	1343 (34.59)	201 (13.15)	<0.001
1	1880 (32.58)	1287 (33.14)	536 (35.08)	
2	1312 (22.74)	699 (18.00)	435 (28.47)	
More than and equal to 3	1033 (17.90)	554 (14.27)	356 (23.30)	
CVD death n (%)	166 (2.88)	92 (2.37)	63 (4.12)	<0.001

*ANOVA was used to test variance across BP trajectories for continuous variables and $\chi 2$ test for categorical variables.
†Number of following chronic diseases during the follow-up period: hypertension, diabetes, coronary heart disease, cardiovascular disease, pulmonary diseases, tuberculosis, cancer, tumor, Parkins
‡Abbreviation: SD Standard Deviation, MAP Mean Arterial Pressure, CVD Cardiovascular Disease.

Supplementary Table S10. **Description of survival status by sex, age group and ethnic group**

**Characteristics**	**CVD mortality**	**Non-cardiovascular mortality**	**Censoring (alive or lost follow-up)**	***P* value***
Sex n (%)				0.334
Female	161 (50.16)	3093 (54.37)	2807 (54.28)	
Male	160 (49.84)	2596 (45.63)	2364 (45.72)	
Age group n (%)†				
<72 years	62 (19.31)	593 (10.42)	2143 (41.44)	<0.001
72-81 years	75 (23.36)	1509 (26.52)	1427 (27.60)	
82-88 years	86 (26.79)	1535 (26.98)	872 (16.86)	
≥89 years	98 (30.53)	2052 (36.07)	729 (14.10)	
Ethnic group‡				<0.001
Han	278 (94.24)	5133 (91.73)	3724 (95.29)	
Other ethnic groups	17 (5.76)	463 (8.27)	184 (4.71)	

*$\chi 2$ test was used to test variance across survival status by sex and age groups.
† Age groups were categorized by the baseline age quantiles of the studied population.
‡ The number was not equal to the total number of the study population because of the missing value on ethnic group.

Supplementary Table S11. Association of blood pressure trajectories on all-cause mortality excluding bedridden participants*

Blood pressure trajectories	All-cause mortality		CVD mortality		Non-CVD mortality	
	HR (95%CI)	*P* value	HR (95%CI)	*P* value	HR (95%CI)	*P* value
**SBP**						
Stable at normal high level	Reference	Reference	Reference	Reference	Reference	Reference
Increasing from normal-low	**0.87 (0.82,0.92)**	**<0.001**	0.82 (0.63,1.06)	0.127	**0.87 (0.82,0.93)**	**<0.001**
Excess SBP trajectory with decreasing trend	**1.35 (1.24,1.47)**	**<0.001**	**1.66 (1.16,2.37)**	**0.006**	**1.33 (1.22,1.45)**	**<0.001**
**DBP**						
Stable at normal high level	Reference	Reference	Reference	Reference	Reference	Reference
Increasing from normal-low	**0.92 (0.87,0.98)**	**0.005**	0.92 (0.70,1.19)	0.512	**0.92 (0.87,0.98)**	**0.006**
Excess SBP trajectory with decreasing trend	**1.16 (1.06,1.26)**	**0.001**	1.29 (0.89,1.88)	0.177	**1.15 (1.05,1.26)**	**0.003**
**PP**						
Stable at normal high level	Reference	Reference	Reference	Reference	Reference	Reference
Increasing from normal-low	**0.89 (0.84,0.94)**	**<0.001**	**0.70 (0.54,0.90)**	**0.006**	**0.90 (0.85,0.96)**	**<0.001**
Excess SBP trajectory with decreasing trend	**1.27 (1.17,1.39)**	**<0.001**	**1.56 (1.07,2.27)**	**0.021**	**1.26 (1.15,1.38)**	**<0.001**
**MAP**						
Stable at normal high level	Reference	Reference	Reference	Reference	Reference	Reference
Increasing from normal-low	**0.90 (0.85,0.96)**	**0.001**	0.84 (0.64,1.10)	0.201	**0.91 (0.85,0.96)**	**0.001**
Excess SBP trajectory with decreasing trend	**1.27 (1.18,1.38)**	**<0.001**	**1.44 (1.03,2.00)**	**0.034**	**1.26 (1.17,1.37)**	**<0.001**

*The fully adjusted model included sex, ethnicity, baseline marriage status, baseline place of residence, main occupation before retirement, average family income, exercise at baseline, smoking status, drinking status, comorbidity status, and frail status.

§Abbreviation: SBP Systolic Blood Pressure, DBP Diastolic Blood Pressure, PP Pulse Pressure, MAP Mean Arterial Pressure, CVD Cardiovascular Disease.

P-value <0.05 was highlighted in bold.

Supplementary Table S12. Association of blood pressure trajectories on all-cause mortality and CVD mortality after multiple imputations*

	**All-cause mortality**		**CVD mortality**		**Non-CVD mortality**	
**Blood pressure trajectories**	**HR (95%CI)**	***P* value**	**HR (95%CI)**	***P* value**	**HR (95%CI)**	***P* value**
**SBP**						
Stable at normal high level	Reference	Reference	Reference	Reference	Reference	Reference
Increasing from normal-low	**0.85 (0.81,0.90)**	**<0.001**	**0.74 (0.58,0.95)**	**0.019**	**0.86 (0.81,0.91)**	**<0.001**
Excess SBP trajectory with decreasing trend	**1.35 (1.24,1.46)**	**<0.001**	**1.95 (1.42,2.67)**	**<0.001**	**1.31 (1.21,1.43)**	**<0.001**
**DBP**						
Stable at normal high level	Reference	Reference	Reference	Reference	Reference	Reference
Increasing from normal-low	**0.89 (0.84,0.94)**	**<0.001**	**0.91 (0.71,1.16)**	0.431	**0.89 (0.84,0.94)**	**<0.001**
Excess SBP trajectory with decreasing trend	**1.15 (1.06,1.25)**	**0.001**	1.24 (0.87,1.76)	0.227	**1.14 (1.05,1.25)**	**0.003**
**PP**						
Stable at normal high level	Reference	Reference	Reference	Reference	Reference	Reference
Increasing from normal-low	**0.88 (0.84,0.93)**	**<0.001**	**0.65 (0.51,0.83)**	**0.000**	0.90 (0.85,0.95)	0.227
Excess SBP trajectory with decreasing trend	**1.31 (1.20,1.43)**	**<0.001**	**2.09 (1.00,2.72)**	**0.000**	**1.27 (1.16,1.39)**	**0.000**
**MAP**						
Stable at normal high level	Reference	Reference	Reference	Reference	Reference	Reference
Increasing from normal-low	**0.88 (0.83,0.93)**	**<0.001**	0.81 (0.63,1.05)	0.109	**0.88 (0.83,0.94)**	**<0.001**
Excess SBP trajectory with decreasing trend	**1.31 (1.20,1.43)**	**<0.001**	**1.66 (1.23,2.24)**	**0.001**	**1.27 (1.17,1.37)**	**<0.001**

*The fully adjusted model included sex, ethnicity, baseline marriage status, baseline place of residence, main occupation before retirement, average family income, exercise at baseline, smoking status, drinking status, comorbidity status, and frail status.

§Abbreviation: SBP Systolic Blood Pressure, DBP Diastolic Blood Pressure, PP Pulse Pressure, MAP Mean Arterial Pressure, CVD Cardiovascular Disease.

P-value <0.05 was highlighted in bold.

Reference

1. Lennon H, Kelly S, Sperrin M, Buchan I, Cross AJ, Leitzmann M, et al. Framework to construct and interpret latent class trajectory modelling. BMJ Open. 2018;8(7):e020683.

2. Nagin DS, Odgers CL. Group-based trajectory modeling in clinical research. Annu Rev Clin Psychol. 2010;6:109-38.

3. Berlin KS, Parra GR, Williams NA. An introduction to latent variable mixture modeling (part 2): longitudinal latent class growth analysis and growth mixture models. J Pediatr Psychol. 2014;39(2):188-203.

4. Shi Z, Zhang T, Byles J, Martin S, Avery JC, Taylor AW. Food Habits, Lifestyle Factors and Mortality among Oldest Old Chinese: The Chinese Longitudinal Healthy Longevity Survey (CLHLS). Nutrients. 2015;7(9):7562-79.

5. Nagin D. Analyzing Developmental Trajectories: A Semiparametric, Group-Based Approach. Psychological Methods. 1999;4:139-57.

6. Berlin KS, Williams NA, Parra GR. An introduction to latent variable mixture modeling (part 1): overview and cross-sectional latent class and latent profile analyses. J Pediatr Psychol. 2014;39(2):174-87.

7. Jones BL, Nagin DS. Advances in Group-Based Trajectory Modeling and an SAS Procedure for Estimating Them. Sociological Methods & Research. 2007;35(4):542-71.

8. Nagin DS. Group-Based Trajectory Modeling: An Overview. In: Piquero AR, Weisburd D, editors. Handbook of Quantitative Criminology. New York, NY: Springer New York; 2010. p. 53-67.

9. Searle SD, Mitnitski A, Gahbauer EA, Gill TM, Rockwood K. A standard procedure for creating a frailty index. BMC Geriatr. 2008;8:24.

10. Chen Q, Tang B, Zhai Y, Chen Y, Jin Z, Han H, et al. Dynamic statistical model for predicting the risk of death among older Chinese people, using longitudinal repeated measures of the frailty index: a prospective cohort study. Age Ageing. 2020;49(6):966-73.

11. Bennett S, Song X, Mitnitski A, Rockwood K. A limit to frailty in very old, community-dwelling people: a secondary analysis of the Chinese longitudinal health and longevity study. Age Ageing. 2013;42(3):372-7.

12. Rockwood K, Mitnitski A. Limits to deficit accumulation in elderly people. Mechanisms of Ageing and Development. 2006;127(5):494-6.
